# Small pouches, but high nicotine doses—nicotine delivery and acute effects after use of tobacco-free nicotine pouches

**DOI:** 10.3389/fphar.2024.1392027

**Published:** 2024-05-22

**Authors:** Nadja Mallock-Ohnesorg, Andrea Rabenstein, Yvonne Stoll, Marcus Gertzen, Benedikt Rieder, Sebastian Malke, Nestor Burgmann, Peter Laux, Elke Pieper, Thomas Schulz, Klaas Franzen, Andreas Luch, Tobias Rüther

**Affiliations:** ^1^ Department of Chemical and Product Safety, German Federal Institute for Risk Assessment (BfR), Berlin, Germany; ^2^ Institute of Legal Medicine, Goethe-University Frankfurt, Frankfurt/Main, Germany; ^3^ Department of Psychiatry and Psychotherapy, LMU University Hospital, LMU Munich, Munich, Germany; ^4^ Department of Psychiatry, Psychotherapy and Psychosomatics, Faculty of Medicine, University of Augsburg, Augsburg, Germany; ^5^ Medical Clinic III, Campus Lübeck, University Hospital Schleswig-Holstein, Lübeck, Germany; ^6^ Airway Research Center North, Member of the German Center for Lung Research (DZL), Großhansdorf, Germany

**Keywords:** nicotine pouches, nicotine delivery, pharmacokinetics, craving reduction, cardiovascular effects, arterial stiffness, high nicotine doses

## Abstract

Tobacco-free nicotine pouches are new nicotine products for oral consumption. They can contain very high nicotine amounts that have not been addressed with clinical studies yet. Thus, nicotine delivery, effects on craving, and side effects were assessed using pouches with up to 30 mg nicotine. In this single-center, five-arm, crossover study, 15 regular cigarette smokers consumed tobacco-free nicotine pouches from different brands with 6, 20, and 30 mg for 20 min. Comparators were nicotine-free pouches and tobacco cigarettes. At baseline and predefined time points over a study period of 240 min, plasma nicotine concentrations, effects on cigarette craving, and side effects were assessed. Cardiovascular parameters including arterial stiffness were measured using a MobilOGraph. Consumption of 30 mg nicotine pouches has led to a higher nicotine uptake compared with the cigarette (C_max_: 29.4 vs 15.2 ng/mL; AUC: 45.7 vs 22.1 ng/mL × h). Nicotine uptake in the acute phase was rapid during use of the 30 mg pouch and cigarette. Extraction rate of nicotine differed between pouches. Use of all products has reduced acute cigarette craving, even the nicotine-free pouch. During consumption of the cigarette and the pouches with 20 and 30 mg, heart rate increased about 27, 12, and 25 bpm, respectively. Parameters for arterial stiffness were elevated and all pouches have induced mouth irritations. The pouches with 30 mg nicotine had overall the strongest side effects and may induce addiction. As craving was also reduced by products with less nicotine, it is questionable whether such high nicotine contents should be allowed on the market. A limit of nicotine content is warranted. The nicotine release rate varies across products and needs to be known to estimate the nicotine delivery.

## 1 Introduction

Smoking increases the risk for several serious diseases such as lung cancer, cardiovascular disease, and chronic obstructive pulmonary disease (National Center for Chronic Disease Prevention and Health Promotion US Office on Smoking and Health, 2014). The combustion of tobacco and the inhalation of the smoke is the primary cause for these smoking-related diseases (National Center for Chronic Disease Prevention and Health Promotion US Office on Smoking and Health, 2014). Cigarette smoke contains not only nicotine but more than 6,500 compounds, some of them hazardous or potentially hazardous (Rodgman and Perfetti, 2013). During the last 5–10 years, several companies introduced tobacco-free nicotine pouches into the United States and many European countries (Robichaud et al., 2020; Mallock et al., 2024). These pouches contain cellulose, nicotine salts, flavors, and acid regulators but no tobacco-leaf material at all (Robichaud et al., 2020; Mallock-Ohnesorg et al., 2023). The pouches are used for durations from 20 to 60 min between lips and gum (Prasad et al., 2022). Nicotine is released and absorbed by the buccal mucosa. A study by one manufacturer analyzed four brands for several compounds such as formaldehyde, acrolein, 1,3-butadiene, benzene, nornitrosonicotine (NNN), and 4-(Methylnitrosamino)-1-(3-pyridyl)-1-butanone (NNK) (Azzopardi et al., 2022a). The concentrations for all these compounds were under the limit of detection (Azzopardi et al., 2022a). In a recent study, the German Federal Institute for Risk Assessment investigated 44 brands of nicotine-containing pouches, covering a range from 1.79 to 47.5 mg nicotine per pouch. Also tobacco-specific nitrosamines were analyzed in these samples, 24 of 44 showed NNN and three of 44 showed NNK above the detection limits of 0.12 ng per pouch (Mallock et al., 2024).

The pharmacokinetic properties are crucial for the development of nicotine addiction (Henningfield and Keenan, 1993; Guimarães et al., 2021). While the highly addictive cigarettes lead to a rapid uptake of nicotine into the blood and consequently into the brain, smoking cessation products such as nicotine patches or gums show a much slower uptake of nicotine (Henningfield and Keenan, 1993; Guimarães et al., 2021). An important question regarding nicotine pouches is whether their pharmacokinetic properties resemble nicotine gums or cigarettes. Up to now, several studies investigated the pharmacokinetic properties of tobacco-free pouches (Lunell et al., 2020; Rensch et al., 2021; Azzopardi et al., 2022b; Chapman et al., 2022; Liu et al., 2022; McEwan et al., 2022). Most studies have been performed by or in association with manufacturers of nicotine pouches, often tobacco companies. Many studies used pouches with nicotine concentrations between 4 and 10 mg per pouch. The application time ranged from 20 to 60 min. Four of six studies compared the pharmacokinetics of pouches with the consumption of a cigarette (Rensch et al., 2021; Chapman et al., 2022; Liu et al., 2022; McEwan et al., 2022) and only four studies included more than one nicotine dose allowing an estimation of dose-dependency (Lunell et al., 2020; Chapman et al., 2022; Liu et al., 2022; McEwan et al., 2022). None of these studies has investigated the pharmacokinetic properties of nicotine pouches with very high nicotine doses.

This five-arm, crossover study was designed to address the data gap on pharmacokinetics of nicotine pouches with high nicotine contents. The primary aim of the study was the comparison of blood plasma nicotine levels following the application of one of four commercially available products, three nicotine containing pouch brands (6, 20, and 30 mg nicotine per pouch), one brand without any nicotine and a cigarette comparator to assess any differences in the nicotine pharmacokinetic profiles over 4 h. The secondary aim of this study was to assess smoking urges and side effects with a focus on cardiovascular effects and local effects at the application site. The findings will help to understand effects of high dose nicotine pouches on addiction and craving reduction in contrast to lower dose products.

## 2 Materials and methods

### 2.1 Aim, study products and ethics

Aim of the study was to assess nicotine uptake, subjective effects, and side effects after use of nicotine pouches with medium to high nicotine contents. Nicotine concentration of venous plasma was determined to assess nicotine uptake in the acute phase (i.e., the first 5 min of consumption) and over the course of 4 h. This single-center, crossover study was conducted with the following five arms:

a. Nicotine-free pouches with mint aroma (Swedish Match, Stockholm Schweden)
b. 6 mg Nicotine pouches with mint aroma (Imperial Brands plc., Bristol, United Kingdom)
c. 20 mg Nicotine pouches with mint aroma (British American Tobacco, London, United Kingdom)
d. 30 mg Nicotine pouches with mint aroma (Fedrs Sp. Z.o.o, Warsaw, Poland)
e. Own-brand tobacco cigarette (different brands).

Pouches were kept between upper lip and gum for 20 min. Products were purchased in October 2021 from online shops. The nicotine strengths mentioned in the manuscript refer to the declared nicotine contents not the analyzed contents. The study was approved by the ethics committee of the LMU Munich (project number 21-0814) and performed in accordance with the principles of the Declaration of Helsinki in the currently valid version. It was registered at the DRKS (DRKS00026244). Informed consent was obtained from all participants before participation in the study. Four hypotheses were tested:

• Nicotine delivery of the studied pouches, measured as C_max_ (maximum plasma concentration) and AUC (area under the plasma-time curve), increases in a dose-dependent manner.
• Use of the pouch with 30 mg nicotine leads to a similarly high plasma nicotine concentration as tobacco cigarettes (C_max_ approximately 15–20 ng/mL).
• In contrast to pouches with 6 mg nicotine, use of pouches with high (20 mg and 30 mg) nicotine doses reduces acute craving for a cigarette comparably to smoking of one cigarette.
• Side effects of pouch use, including cardiovascular effects (i.e., heart rate, arterial stiffness), increase in a dose-dependent manner.

### 2.2 Participants

The single-center, five-arm, crossover study included 15 active smokers. Recruitment of participants took place via advertisement (social media, LMU intranet, LMU newsletter). Enrolled participants that fulfilled inclusion and exclusion criteria gave written informed consent. Inclusion criteria were: Age between 18 and 55 years, active smoking for at least 5 years with more than 10 cigarettes per day, 12 h of abstinence from any nicotine product prior to testing, CO levels < 5 ppm (in the expiratory air analyzed using a micro-smokerlyzer; Bedfont Scientific Ltd., Anif, Austria) and plasma nicotine concentration at baseline < 10 ng/mL to verify abstinence from cigarettes and other nicotine products, and the ability to give consent. Exclusion criteria were: Age under 18 or over 55 years, use of other nicotine products (e.g., nicotine pouches, snus, e-cigarettes) more often than once a week, acute psychiatric illness according to ICD-10/DSM IV or other serious psychiatric disorders, acute suicidality, pregnancy, breastfeeding, current abuse of drugs, medication, or alcohol, malignant cancer in the past 5 years, serious internal illness, especially cardiovascular diseases, such as manifest arterial hypertension, severe heart disease (DCM, history of heart attack), pacemaker implantation, respiratory diseases (e.g., respiratory failure, asthma, COPD), and severe active infectious disease.

### 2.3 Study design

**FIGURE 1:**
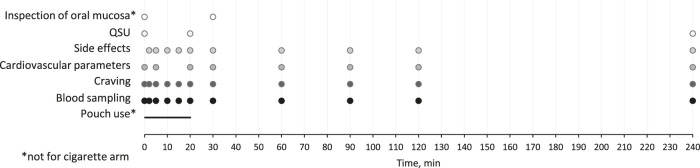
Study design with measurements and their time points.

The study was conducted from September 2021 to May 2022 at LMU University Hospital in Munich, Germany with six visits per participant. At the first visit, participants were screened for inclusion and exclusion criteria, sociodemographic data and smoking behavior in the past 30 days were inquired, and physical dependence for cigarettes was assessed using the Fagerström Test for Cigarette Dependence (FTCD) (Heatherton et al., 1991). The following visits were study days. While at the first study day, all participants consumed their own-brand cigarette, the order of pouches (with 0, 6, 20, or 30 mg nicotine) used at study days 2–5 was randomized. Prior to study days, participants were asked to stay abstinent from any nicotine product for at least 12 h. The time line at study days with the measurements taken is presented in Figure 1.

Participants were instructed either to smoke one of their own-brand cigarettes as they usually do or to place the pouch between upper lip and gum and keep it for 20 min without chewing or sucking it. 30 min before, during, and 30 min after pouch use, participants were asked not to use chewing gum, eat any food, drink more than small amounts of water, and brush their teeth. Over the whole observation period of 240 min, participants were asked not to use any other further nicotine product or consume food or beverages containing caffeine, mint, or licorice. The abstinence was considered to avoid possible interferences with nicotine metabolism, subjective effects (e.g., head buzz), or cardiovascular effects (Benowitz et al., 2009; Deutch et al., 2019).

### 2.4 Measurements

Venous blood was sampled using peripheral venous Safety Multifly cannulas and S-Monovettes (Sarstedt AG & Co. KG, Nümbrecht, Germany) at baseline and at 2, 5, 10, 15, 20, 30, 60, 90, 120, and 240 min and was cooled until preparation for quantitative nicotine analysis as described below. Smoking urges were assessed at baseline, at 20 and at 240 min using the German version of the Questionnaire on Smoking Urges (QSU-G) (Müller et al., 2001). Acute craving after a cigarette was asked to be rated (“I now feel the urge for a cigarette”) at baseline and at 2, 5, 10, 15, 20, 30, 60, 90, 120, and 240 min on a seven-point Likert scale from 1 (not at all true) to 7 (completely true).

Cardiovascular parameters were measured using a Mobil-O-Graph (I.E.M. GmbH, Stollberg, Germany) at baseline and at 2, 5, 20, 30, 60, 90, 120, and 240 min. The parameters heart rate, peripheral and central blood pressure, augmentation index adjusted at HR 75 bpm (AIX@75), and total peripheral resistance/vascular resistance (TVR) were measured and calculated with the Mobil-O-Graph software version HMS CS 4.2 (I.E.M. GmbH, Stollberg, Germany). The instrument and measurement procedure are described elsewhere (Wassertheurer et al., 2010; Weber et al., 2011; Hauck et al., 2023).

The side effects head buzz, mouth or throat irritations, lightheadedness, dizziness, cold hands or feet, palpitations, headache, perspiration, nausea, and urge to vomit were assessed on a numeric rating scale (NRS) from 0 (no effect) to 10 (strongest effect) at 2, 5, 10, 15, 20, 30, 60, 90, 120, and 240 min. Salivation was inquired on a scale from 0 = lowest salivation (dry mouth) over 5 = normal salivation to 10 = highest salivation (hypersalivation). At study days at which a pouch was used, the oral mucosa was inspected for redness or ulceration at baseline and at 30 min.

### 2.5 Analysis of nicotine, cotinine, and trans-3′-hydroxycotinine from blood samples

Whole blood was centrifuged (1,500 g, 10 min, 4°C) and 10 µL internal standard mix (500 ng/mL nicotine-d_3_, cotinine-d_3_, hydroxycotinine-d_3_ in acetonitrile) was added to 990 µL blood plasma. Samples were stored at LMU University Hospital in Munich at −80°C and shipped to BfR in Berlin on dry ice. A previously described validated method was used for the quantification of nicotine, cotinine, and trans-3′-hydroxycotinine (hydroxycotinine) from plasma using protein precipitation and liquid chromatography—tandem mass spectrometry (LC-MS/MS) with a matrix-matched calibration (Mallock et al., 2021).

### 2.6 Determination of nicotine extraction from pouches

After removal, pouches were individually wrapped and stored at −20°C before they were shipped on dry ice to BfR in Berlin, Germany. Method for nicotine content determination was modified from a previously described method (Mallock et al., 2024). Pouches were weighted and placed into an Erlenmeyer flask with stopper followed by a liquid-liquid extraction using 10 mL ultra-pure water, 5 mL sodium hydroxide solution (2 M), and 20 mL n-hexane with the internal standard n-hexadecane (0.5 g/L) for 75 min at 350 rpm (orbital shaker GFL 3005, Lauda-GFL, Lauda-Königshofen, Germany). Of the organic phase, 2 µL were injected into the GC/FID system (G1530A series from Agilent Technologies/Hewlett Packard, Agilent Technologies, Waldbronn, Germany) and analyzed as described in the Supplementary Material.

### 2.7 Pharmacokinetic (PK) parameters and statistics

For calculation of the half-life (t_1/2_), the elimination rate constant (i.e., the slope of the terminal elimination phase) was determined using the last two nicotine plasma concentrations (at 120 and 240 min). The plasma nicotine concentrations determined at baseline and the individual elimination rate constant were used to determine the residual nicotine concentration at the subsequent nicotine sampling times. These values were then subtracted from the subsequent nicotine levels before PK parameters were calculated. Areas under the plasma concentration-time curve (AUC) were calculated with the linear trapezoid rule. The highest plasma nicotine concentration per curve was used as C_max_ and the according time point for t_max_. Delays in blood sampling were noted and considered when the individual AUCs were calculated. Relative bioavailability (F_rel_) between two pouches was calculated using the following equation with the analyzed nicotine content as dose (D):

$$F_{rel}=100∙\frac{{AUC}_{\text{Pouch}\;1}∙D_{\text{Pouch}\;2}}{{AUC}_{\text{Pouch}\;2}∙D_{\text{Pouch}\;1}}$$

Nicotine metabolite ratio (NMR) was calculated by dividing hydroxycotinine by cotinine plasma concentrations at baseline at the first study day. NMR can be used as a surrogate for CYP 2A6 activity (Dempsey et al., 2004; Allenby et al., 2016) with low values (NMR < 0.31) for slow metabolizers and higher values (NMR > 0.31) for normal or rapid metabolizers (Lerman et al., 2015). Median and interquartile ratios (IQR) were calculated for participant characteristics including NMR; for t_max_, median and range. For AUC and C_max_, geometric means and coefficients of variation (CV) were calculated. Arithmetic means and 95% confidence intervals (CI) were used for mean plasma curves. Statistical Package for Social Sciences (SPSS) version 26.0 was used for statistical analysis. Two-sided paired t-tests were applied to evaluate differences between groups. For C_max_ and AUC, lognormal values were used. Baseline mean values were used as statistical references for the cardiovascular parameters blood pressure, heart rate, and arterial stiffness parameters. Cardiovascular parameters were tested for normal distribution by Kolmogorov-Smirnov tests and a two-way repeated measures ANOVA based on baseline measurements was used to estimate for an interaction between the product used and time. To individually analyze differences at the various time points in between the study arms, ANOVA is used.

## 3 Results

### 3.1 Participants

Of the 18 recruited participants, three dropped out without completion of all study arms. The characteristics of the 15 participants that completed the study are summarized in Table 1. Individual characteristics are presented in Supplementary Table 1. Participants had a low to moderate physical cigarette dependence as measured with the FTCD. Eleven participants had an NMR > 0.31 and were classified as normal/rapid metabolizers; four were classified as slow metabolizers (participants 5, 7, 11, and 15). The participants were daily smokers who smoked about 12 cigarettes per day.

**TABLE 1:** Participant characteristics including smoking behavior in the past 30 days.

Age, median (IQR)	30 (24–40)
Sex, female, *n* (%)	8 (53%)
Sex, male, *n* (%)	7 (47%)
Height in cm, mean (SD)	171.8 ± 6.0
Weight in kg, mean (SD)	78.1 ± 17.5
Body mass index (BMI), mean (SD)	26.4 ± 5.5
Fagerström Test for Cigarette Dependence (FTCD), median (IQR)	4 (3–5)
Nicotine Metabolite Ratio (NMR), median (IQR)	0.44 (0.31–0.63)
Number of days cigarettes were smoked within the last 30 days, median (IQR)	30 (30–30)
Number of cigarettes smoked on a day with smoking, median (IQR)	12 (12–15)

IQR, interquartile ratio; SD, standard deviation.

### 3.2 Nicotine delivery and nicotine extraction from pouches

**FIGURE 2:**
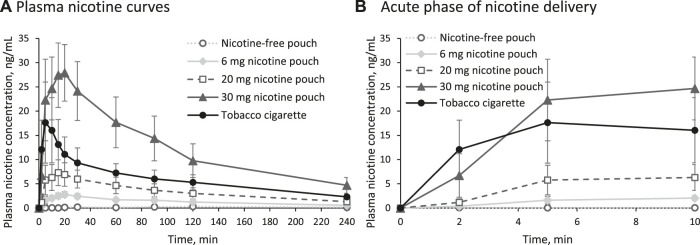
**(A)** Plasma nicotine curves for the five study arms and **(B)** magnification of the acute phase (arithmetic means and 95% confidence interval).

Mean plasma nicotine curves during and after consumption of the study products are presented in Figure 2A. Individual plasma nicotine curves are displayed in Supplementary Figure 1 and the individual nicotine concentrations are shown in Supplementary Tables 2–6. Consumption of nicotine-free pouches did not result in a nicotine uptake. A magnification of the acute phase, meaning the first minutes of consumption, is shown in Figure 2B. Rise of plasma nicotine levels was fastest for 30 mg nicotine pouches and tobacco cigarettes compared with the other tested products.

The PK parameters C_max_, t_max_, AUC_0–240min_, and t_1/2_ are summarized in Table 2 for the nicotine-containing products. The parameters C_max_ and AUC_0–240min_ increased in the order 6 mg nicotine pouch < 20 mg nicotine pouch < tobacco cigarette < 30 mg nicotine pouch with statistically significant differences between all nicotine pouches (C_max_: all *p*-values < 0.0001; AUC_0–240min_: all *p*-values < 0.0001). In comparison with the tobacco cigarette, *p*-values for C_max_ were 0.0000001, 0.001, and 0.0007 for pouches with 6, 20, and 30 mg nicotine, respectively. For AUC_0–240min_, these values were 0.0000001, 0.001, and 0.0002. With tobacco cigarettes, t_max_ was reached the fastest in line with the shorter consumption duration. The half time t_½_ of nicotine was calculated showing no differences between study arms.

**TABLE 2:** Summary of relevant pharmacokinetic parameters for the nicotine containing study products and mean extraction rate of nicotine from pouches.

Product	C_max_ (ng/mL)	t_max_ (min)	AUC_0–240min_ (ng/mL × h)	t_1/2_ (h)	Nicotine extraction (%)
6 mg nicotine pouch	2.8 (39%)	20 (5–36)	4.9 (65%)	2.4 (1.2)	38%
20 mg nicotine pouch	7.1 (72%)	15 (5–60)	11.6 (77%)	2.2 (1.5)	24%
30 mg nicotine pouch	29.4 (58%)	15 (5–30)	45.7 (60%)	1.8 (0.6)	52%
Tobacco cigarette	15.2 (111%)	5 (2–15)	22.1 (59%)	2.3 (1.4)	—

C_max_ and AUC: Geometric mean and coefficient of variation (CV%); t_max_: Median and range; t_1/2_: arithmetic mean and standard deviation.

Nicotine content of unused nicotine pouches were analyzed with GC/FID. Analyzed nicotine contents were 4.8 ± 0.4 mg, 16.3 ± 3.1 mg, and 27.1 ± 0.2 mg for the nicotine pouches declared with 6, 20, and 30 mg, respectively. Remaining nicotine in used pouches was analyzed (see Supplementary Table 7) and rates of nicotine extraction were calculated. Mean nicotine extraction rates differed between pouches as shown in Table 2. Considering the analyzed total content and the remaining nicotine contents, mean nicotine doses extracted were 1.8 ± 0.8 mg, 4.7 ± 3.5 mg, and 14.1 ± 3.0 mg for the pouches with a nominal nicotine content of 6, 20, and 30 mg, respectively. Relative bioavailability in relation to the 6 mg (analyzed 4.8 mg) pouch was 70% for the 20 mg (analyzed 16.3 mg) pouch and 165% for the 30 mg (analyzed 27.1 mg) pouch.

### 3.3 Effects on craving

**FIGURE 3:**
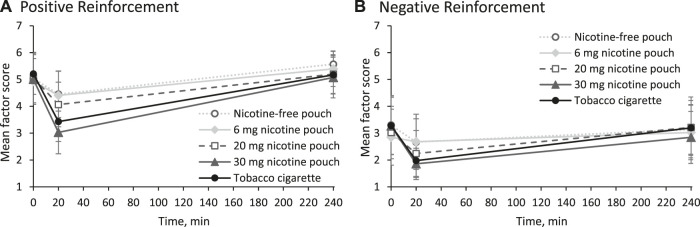
Mean scores and 95% confidence intervals for **(A)** factor 1 (positive reinforcement) and **(B)** factor 2 (negative reinforcement) of urges to smoke.

Effects on smoking urges were measured using the QSU-G at baseline, at 20 min, and at 240 min as presented in Figure 3. Positive reinforcement is described by factor 1 (Figure 3A) and reflects, for example, the anticipation of positives effects from smoking. Mean score for factor 1 was significantly reduced by tobacco cigarette and by the pouches with 20 and 30 mg nicotine at 20 min (*p* < 0.01). Negative reinforcement, described by factor 2 (Figure 3B), reflects, for example, the anticipation of relief from withdrawal. Mean score for factor 2 was significantly reduced by tobacco cigarette and by the pouches with 20 and 30 mg nicotine at 20 min (*p* < 0.05).

**FIGURE 4:**
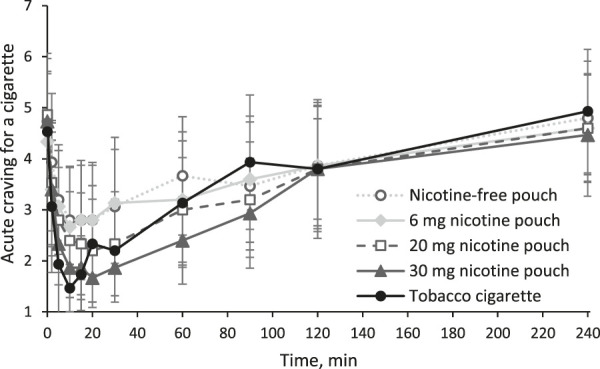
Acute craving for a cigarette inquired with a single question (“I now feel the urge for a cigarette”) answered on a scale from 1 (lowest) to 7 (highest) as mean scores and 95% confidence intervals.

Additionally, acute cigarette craving was inquired with a single question (“I now feel the urge for a cigarette”) at each time point of blood sampling as shown in Figure 4. All products, even the nicotine-free pouch, led to a statistically significant reduction of acute craving for a cigarette (all *p* values < 0.05, see Supplementary Material). In agreement with the earlier time point for t_max_, reduction was fastest for the tobacco cigarette. Difference in craving reduction was statistically significant between nicotine-free pouch and 30 mg nicotine pouch (*p* = 0.04). Individual ratings for acute craving are presented in Supplementary Tables 8–12.

### 3.4 Cardiovascular effects and arterial stiffness

**FIGURE 5:**
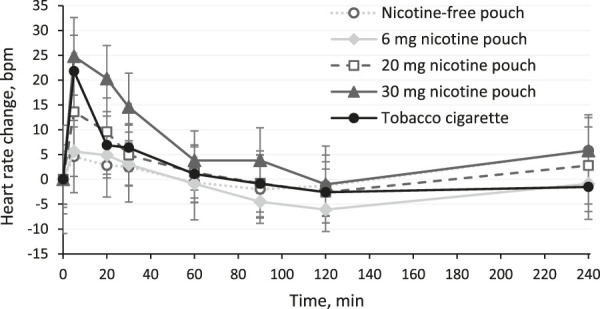
Changes in heart rate (mean and 95% confidence interval).

Changes in heart rate are displayed in Figure 5. Increases in heart rate occurred in the beginning of product use (5 min) for the 20 and 30 mg nicotine pouches, tobacco cigarettes, and slightly for the 6 mg nicotine pouches. Increases were the strongest for the 30 mg pouches and tobacco cigarettes. Effects on systolic and diastolic blood pressure were minor for all study arms as summarized in the Supplementary Tables 13–15.

Besides peripheral and central blood pressure, the parameters of arterial stiffness, augmentation index adjusted at HR 75 bpm (AIX@75) and total peripheral resistance/vascular resistance (TVR), were measured at baseline and at 5, 20, 30, 60, 90, 120, and 240 min. Besides peripheral systolic blood pressure, there was a significant increase also in central systolic blood pressure in the study arms using pouches with high nicotine concentrations and tobacco cigarette in the acute phase, the beginning of product consumption. Further, there was a significant increase in the parameters of arterial vascular stiffness. AIX@75 and TVR were significantly elevated during consumption of the high nicotine pouches (20 and 30 mg) and tobacco cigarette. Results are shown in the Supplementary Tables 16, 17.

### 3.5 Other side effects

**FIGURE 6:**
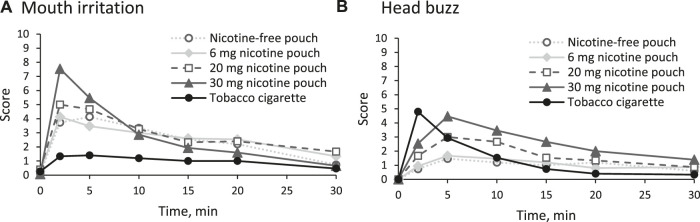
Reported side effects **(A)** mouth irritation and **(B)** head buzz in the first 30 min on a numeric rating scale (NRS) from 0 (no effect) to 10 (strongest effect).

No serious adverse effects were reported. One participant who used the 30 mg nicotine pouch experienced circulation problems with mild symptoms. By employing general measures such as elevating the legs, the participant’s condition normalized within a few minutes and no serious adverse event was reported.. Strong mouth irritations were reported in the first 10 min of use of the 30 mg nicotine pouch (Figure 6A). Use of the other pouches resulted in medium mouth irritations, regardless of the nicotine strength, while cigarette smoking did not have such an effect. Reported head buzz (i.e., the feeling of a slight intoxication) peaked in the cigarette arm at 2 min and in the pouch arms at 5 min. Cigarette smoking and use of the 30 mg pouch led to a head buzz with medium effect size. None of the other inquired side effects (throat irritations, lightheadedness, dizziness, cold hands or feet, palpitations, headache, perspiration, nausea, and urge to vomit) was rated higher than 3 out of 10 at any time point as summarized in Supplementary Table 18. For salivation, effects in both directions (less salivation or more salivation than usual) were monitored. No increased salivation was observed and a slightly drier mouth was reported, especially in the tobacco cigarette arm (see Supplementary Table 19).

The oral mucosa was inspected before and 10 minutes after pouch use for redness or ulceration. A slightly increased redness was revealed in 1, 4, 3, and 5 cases after use of the pouches with 0, 6, 20, and 30 mg nicotine, respectively. An ulceration was visible in one case after use of the 6 mg pouch. In the remaining cases, appearance of the oral mucosa did not change compared with the inspection at baseline.

## 4 Discussion

In the landscape of alternative nicotine delivery systems (ANDS), nicotine pouches are among the most recent products. Where available, they are mostly not adequately addressed with specific regulations regarding, for example, the nicotine content (Duren et al., 2023). Scientific knowledge on these products is scarce and was predominantly generated by the manufacturers of the products. In previously published clinical trials, only pouches up to 10 mg nicotine were studied (Lunell et al., 2020; Rensch et al., 2021; Azzopardi et al., 2022b; Chapman et al., 2022; Liu et al., 2022; McEwan et al., 2022). However, much higher nicotine contents are available, up to 50 mg were previously reported (Mallock et al., 2024). The herein presented clinical trial is the first to report nicotine pharmacokinetics, subjective effects, and side effects from use of nicotine pouches with 20 and 30 mg. Additionally, this work is the first to include nicotine-free oral pouches as a control. Nicotine content of the pouches were analyzed with GC/FID and the pouches only contained between 80% and 90% of the declared nicotine content. For brevity, the declared nicotine content is used throughout this manuscript.

An overview over results from previous pharmacokinetic studies is given in Table 3 including highest studied nicotine strength, duration of use, highest achieved mean C_max_, and highest achieved mean AUC. Mean C_max_ after 20 min of use of the 30 mg nicotine pouch was much higher compared with the highest mean C_max_ values achieved in one of the other studies after the use of pouches with up to 10 mg nicotine regardless of the use duration. In three studies, the participants kept the products for 60 min in their oral cavities (Lunell et al., 2020; Azzopardi et al., 2022b; McEwan et al., 2022), which is three times the use duration compared with the presented study. A use duration of 20 min was chosen for two reasons: Firstly, a market survey conducted by a product manufacturer has revealed that the preferred duration for holding nicotine pouches in the oral cavity ranges from 5 to 20 min in Germany (Prasad et al., 2022). The higher the nicotine strength of the pouch, the shorter was the preferred hold time (Prasad et al., 2022). Secondly, as no clinical data for the use of pouches with high nicotine concentrations were available, it was unclear how participants would react to such a high nicotine dose. Accordingly, the duration time was kept relatively short and nicotine doses above 30 mg were not included. However, as seen in Table 2, participants only extracted 52% (14 ± 3.0 mg) of the nicotine contained in the 30 mg pouches and even less from the other pouches. It is possible that with a longer use duration, more nicotine would have been extracted potentially leading to increased side effects. In two other studies, nicotine extraction rates have been analyzed, both with use durations of 60 min. Lunell et al. have reported mean extraction rates of 56%, 59%, and 50% for pouches with 3, 6, and 8 mg nicotine, respectively (Lunell et al., 2020). In the study by Azzopardi et al., a mean of 62% was extracted from the 4 mg pouch (Azzopardi et al., 2022b). The total delivered nicotine, represented by the AUC, depends on the administration time of nicotine. Accordingly, the AUC was higher in two studies with a lower nicotine dose but a longer duration of use (Table 3). It should be noted that the last blood sampling time point (t) has a large influence on the AUC_0–t_ and that it varied across the studies mentioned in Table 3. Thus, the different AUC_0–t_ values should be compared with caution.

**TABLE 3:** Overview over published clinical data on nicotine pouch pharmacokinetics.

Study (name, year)	Highest studied nicotine strength (mg)	Duration of use (min)	Highest mean C_max_	Highest mean AUC_0-t_	Last blood sampling time point (t) (h)
Presented study	30	20	29.4 ng/mL	45.7 ng/mL × h	4
Lunell et al. (2020)	8	60	18.5 ng/mL	58.4 ng/mL × h	6
McEwan et al. (2022)	10	60	18.4 ng/mL	53.7 ng/mL × h	6
Rensch et al. (2021)	4	30	12.1 ng/mL	19.5 ng/mL × h	3
Chapman et al. (2022)	10	20	7.9 ng/mL	18.4 ng/mL × h	8
Azzopardi et al. (2022b)	4	60	8.3 ng/mL	29.7 ng/mL × h	12
Liu et al. (2022)	8	30	14.5 ng/mL	24.0 ng/mL × h	3

In the presented study, mean C_max_ and AUC were significantly higher after use of the 30 mg nicotine pouches compared with tobacco cigarettes. In addition to nicotine delivery, assessing the nicotine flush during the acute phase of consumption is crucial for evaluating the product’s risk. As seen in Figure 2B, use of the 30 mg nicotine pouch has led to a similarly fast nicotine uptake compared with cigarette smoking. The rate at which nicotine concentration increases in the bloodstream and, consequently, in the brain is associated with the activation of the reward system and the product’s addictive potential (Henningfield and Keenan, 1993). The highly addictive nature of the tobacco cigarette can serve as a reference point. A similarly rapid nicotine uptake during the acute phase may suggest a comparable level of addictiveness for the alternative product. However, addictiveness of a product cannot be ruled out only by demonstrating a slower nicotine uptake. Tobacco dependence is complex and involves many behavioral factors as for instance (sensory) conditioning or social learning (Brandon et al., 2004; Eissenberg, 2004; Glautier, 2004).

It should also be noted that inhaling cigarette smoke results in a more rapid increase in nicotine levels in arterial blood compared to venous blood (Henningfield et al., 1993). Nicotine is transported to the brain by arterial blood. Therefore, venous blood concentrations are a poorer surrogate for post smoking nicotine concentrations in the brain than arterial samples. Due to the buccal resorption, the distribution between arterial and venous blood in the early phase of nicotine pouch use is likely to be different to cigarette use. Thus, it remains to be studied how venous plasma concentrations translate to nicotine levels in the brain in the context of nicotine pouch consumption. As an approximation for nicotine effects in the brain, the participants were asked to rate the sensation of a head buzz at different time points. During cigarette smoking, the peak sensation was achieved immediately at the first assessment point after 2 min. During use of the 30 mg nicotine pouch, head buzz peaked after 5 min much before t_max_. The peak effect sizes of head buzz during cigarette or 30 mg nicotine pouch consumption were comparable.

Another aspect related to the nicotine delivery is the reduction of craving for a cigarette. All participants were regular smokers with mild to moderate addiction according to their FTCD score. Nicotine products that efficiently reduce craving in concert with a markedly lower exposure to harmful chemicals can be beneficial for an addicted smoker who is unable to overcome nicotine use (Kozlowski and Abrams, 2016; Hatsukami and Carroll, 2020). While the toxicity of cigarette smoke, which contains over a hundred highly toxic chemicals, has been extensively studied, much less is known about nicotine pouches. However, when considering recent independent studies (Mallock-Ohnesorg et al., 2023; Mallock et al., 2024), it can be expected that nicotine pouches lead to a substantially lower exposure to toxicants compared to cigarette smoke. All tested products, including the nicotine-free pouch, have significantly reduced acute cravings for a cigarette. During cigarette smoking and consumption of the 20 and 30 mg nicotine pouches, the lowest mean score for acute craving was with 20 min shortly after t_max_ at 15 min. Consumption of the pouches with 0 and 6 mg reduced craving for a cigarette in the first 10 minutes. This underlines that factors such as sensory cues or expectation of reward play a role in reducing craving by these oral products. The acute craving increased most rapidly in the cigarette arm following the initial satisfaction. Craving reduction was not statistically different between the tobacco cigarette and both pouches with high nicotine contents, 20 and 30 mg. Additional to the question on acute craving for a cigarette, the QSU was answered at three time points, at baseline, at 20 min, and at 240 min. With the QSU, effects on positive reinforcement factors (e.g., expectation of a positive effect from smoking) and negative reinforcement factors (e.g., expectation of relief from withdrawal symptoms) for cigarette smoking were measured with multiple items. Both factors of smoking urges were reduced by the tobacco cigarette and the nicotine pouches with high nicotine contents, 20 and 30 mg. As participants did not answer the QSU at 10 min, early effects in craving reduction by the 0 and 6 mg pouches with the single-item measurement were not assessed.

In terms of side effects, cardiovascular effects were expected to be triggered by nicotine. Nicotine stimulates the acetylcholine-receptors causing reaction in the central nervous and vegetative nerval system with a consecutive increasing heart rate and blood pressure (Benowitz and Burbank, 2016). Indeed, in the early phase of consumption, 30 mg nicotine pouches and tobacco cigarettes led with an increase of approximately 25 bpm to similarly strong rises of heart rate. The 20 mg nicotine pouches led to a lower rise, while the 6 mg pouch increased heart rate only slightly and the nicotine-free pouches did not affect heart rate significantly. Only two of the previously published clinical studies have monitored cardiovascular effects of nicotine pouch use (Lunell et al., 2020; Chapman et al., 2022). Chapman et al. have not reported any changes in heart rate or blood pressure after use of a 10 mg nicotine pouch with a C_max_ of 7.9 ng/mL (Chapman et al., 2022). Lunell et al. have described an increase of 10.5 bpm after 60 min of use of the 6 mg nicotine pouch with a C_max_ of 14.7 ng/mL (Lunell et al., 2020). This underlines the dose-dependency of the acute effects of nicotine on heart rate from nicotine pouches.

In addition to heart rate and blood pressure, parameters to measure arterial stiffness (AIX@75, TVR) were assessed. Significant effects were found especially for the high dose pouches as well as the combustible cigarette at the first time point of measurement, at 5 min. Since our study has only a relatively short follow-up period of 240 min, long-term statements can only be formulated speculatively. Most of the variations in central and peripheral blood pressure, heart rate, and arterial stiffness parameters can be attributed to nicotine. Cigarette smoking is associated with a substantially increased risk of cardiovascular disease and mortality (Benowitz and Liakoni, 2022). The main contributors are not nicotine but combustion products that induce chronic inflammation and cardiovascular dysfunction (Benowitz and Liakoni, 2022). Users of snus, a type of oral smokeless tobacco, do not show an increased risk of cardiovascular disease compared to never smokers, but the risk for fatal outcomes is elevated (Benowitz and Liakoni, 2022). Looking at Sweden where the male population predominantly uses snus rather than cigarettes, the cardiovascular health in men has improved over the last decades compared with other developed countries or with Swedish women (Foulds et al., 2003). As nicotine pouches are a very similar product as snus, it is likely that they also pose a much lower risk for cardiovascular events than cigarette smoking does. However, considering the acute effects on parameters reflecting arterial stiffness, an increased risk for arterial hypertension, atherosclerosis, or myocardial infarction especially for consumers with an already existing cardiovascular disease is possible.

Besides cardiovascular effects, local effects were of special interest. The oral mucosa was inspected 10 minutes after the pouches were removed. In some cases, an increased redness was visible and in one case, after use of the 6 mg pouch, an ulceration was detected. Moderate mouth irritation was reported by the participants at the beginning of consumption of the pouches with 0, 6, and 20 mg nicotine. The 30 mg nicotine pouches induced a strong mouth irritation, while the mouth irritation during smoking was low. This suggests a tendency that nicotine may contribute to local effects. However, as the pouches with no nicotine or low amounts also induced local adverse effects, other substances are involved. This is in line with an *in vitro* toxicity study of nicotine pouch extracts in oral fibroblasts in which cytotoxic effects were found to be independent from the nicotine dose (Rinaldi et al., 2023). Results of another *in vitro* cytotoxicity study in gingival epithelial cells also indicate that nicotine pouches can have adverse local effects (Shaikh et al., 2022).

One industry study has compared nicotine deliveries of nicotine pouches with similar nicotine contents (8–10 mg) but from different brands (McEwan et al., 2022). Their findings suggest that there is no direct correlation between the nicotine content of pouches and the nicotine delivered to the bloodstream. Relative bioavailability (regarding C_max_) in relation to the product with the lowest release ranged from 137% to 245% (McEwan et al., 2022). Results of the herein presented study also speak against a linear relationship between nicotine delivery and nicotine content in the pouch, visible in the relative bioavailability ranging from 70% to 165%. Also, the C_max_ does not increase proportionally with the nicotine content. This is likely due to different nicotine releases from the pouches. The variability in residual nicotine proportions post-use indicates that different products release varying percentages of their total nicotine content. This varied from 24% for the 20 mg nicotine pouch to 52% for the 30 mg nicotine pouch. Lunell et al. have investigated three different nicotine strengths from the same brand and the C_max_ increase was almost linear in relation to the nicotine content (Lunell et al., 2020). *In vitro* nicotine release studies confirm that nicotine pouches can have different release rates and different release profiles depending on the formulation (Aldeek et al., 2021). This should be considered when interpreting the results from the 6 and 20 mg nicotine pouch arms of this study. The 20 mg nicotine pouch chosen for this study happened to only release 24% of its nicotine under the given use conditions. With a different formulation, nicotine pouches can release a higher percentage of nicotine. This means that higher plasma nicotine levels are possible during use of other 20 mg nicotine pouches.

## 5 Limitations

The participants of this study were not experienced users of oral tobacco/nicotine products. Experienced users of such products may have responded with different subjective effects or may have used the products differently. Although oral products do not allow the same degrees of freedom in terms of use as products for inhalation do (i.e., multiple puffing parameters), some parameters (e.g., the insalivation of the pouches) can be adjusted to manipulate nicotine release. However, enlisting regular users was not possible due to the low prevalence of regular oral tobacco/nicotine product use in Germany. The number of participants was too low for an in-depth statistical analysis investigating potential influences of participant characteristics, e.g., the metabolizer status. Another limitation is that the study only investigated a limited selection of products with its five study arms. Additionally, nicotine contents even higher than the 30 mg used in this study are available. Considering the great variability of nicotine release rates, more data on nicotine deliveries of pouches with high nicotine contents (i.e., 20 mg nicotine and more) is needed. All products come from different brands. This design was chosen in order to cover different formulations and subsequently different nicotine releases. Consequently, the results of this study including differences in subjective effects and side effects may have been affected by other constituents than nicotine as well.

## 6 Conclusion

The presented study is the first to investigate the use of nicotine pouches with high nicotine contents of up to 30 mg. The nicotine delivery of the herein used 30 mg nicotine pouches exceeded the nicotine delivery of a tobacco cigarette. Overall, these pouches with 30 mg nicotine had the strongest side effects. This study is also the first to include nicotine-free pouches and thus has a control arm with a product that comes close to a placebo. It was shown that use of nicotine-free pouches reduced cigarette craving and induced side effects such as mouth irritation. Consequently, other factors, for example, sensory aspects or expectations of reward, play an important role. Considering the craving reduction by pouches with no or low nicotine content, it is questioned whether nicotine pouches with high nicotine contents such as 30 mg are needed to provide addicted smokers with an alternative for cigarettes. The presented data also demonstrate that knowledge of only the nicotine content is not enough to estimate the nicotine delivery of a product. Further research, e.g., long-term use studies, are needed to clarify the nicotine content that is actually needed to provide an appropriate alternative for smokers. Ideally, it is as low as possible to reduce addictive potential and cardiovascular side effects. Whether nicotine pouches can pose an alternative for cigarette smoking is possible but yet unclear. However, the presented data suggest that nicotine pouches with very high nicotine doses are likely to induce addiction. Therefore, it is advisable that the nicotine content of pouches is limited and more information such as nicotine release rate are available to allow consumers to make informed choices.

## Data Availability

The original contributions presented in the study are included in the article/Supplementary Material, further inquiries can be directed to the corresponding author.
