# Supplementary material for: Small pouches, but high nicotine doses—nicotine delivery and acute effects after use of tobacco-free nicotine pouches
Source: Front Pharmacol. 2024 May 22;15:1392027. doi: 10.3389/fphar.2024.1392027 (PMC11150668; doi:10.3389/fphar.2024.1392027)
Supplement: Supplementary file 1 [file DataSheet1.docx]

**Supplementary Material**

Small pouches, but high nicotine doses - Nicotine delivery and acute effects after use of tobacco-free nicotine pouches

Nadja Mallock-Ohnesorg^1, 2,*^, Andrea Rabenstein^3^, Yvonne Stoll^3^, Marcus Gertzen^4^, Benedikt Rieder^3^, Sebastian Malke^1^, Nestor Burgmann^3^, Peter Laux^1^, Elke Pieper^1^, Thomas Schulz^1^, Klaas Franzen^5,6^, Andreas Luch^1^, Tobias Rüther^3^

^1^German Federal Institute for Risk Assessment (BfR), Department of Chemical and Product Safety, 10589 Berlin, Germany

^2^Institute of Legal Medicine, Goethe-University Frankfurt, Kennedyallee 104, 60596 Frankfurt/Main, Germany

^3^Department of Psychiatry and Psychotherapy, LMU University Hospital, LMU Munich, 80336 Munich, Germany

^4^Department of Psychiatry, Psychotherapy and Psychosomatics, Faculty of Medicine, University of Augsburg, 86156 Augsburg, Germany

^5^Medical Clinic III, Campus Lübeck, University Hospital Schleswig-Holstein, 23562 Lübeck, Germany

^6^Airway Research Center North, Member of the German Center for Lung Research (DZL), 22927 Großhansdorf, Germany

*corresponding author: Mallock-Ohnesorg@med.uni-frankfurt.de

Table of contents

[1. Participant characteristics 2](#_Toc153745646)

[2. Individual plasma nicotine curves 3](#_Toc153745647)

[3. Craving 7](#_Toc153745648)

[4. Cardiovascular effects and arterial stiffness 9](#_Toc153745649)

[5. Side Effects 12](#_Toc153745650)

[6. Gas chromatography method for nicotine determination 15](#_Toc153745651)

# 1. Participant characteristics

Participant characteristics assessed at the first study day are summarized in the main text. Individual values are presented in Supplementary Table 1.

**Supplementary Table 1.** Parameters age (in years), sex (f: female; m: male), height (in cm), weight (in kg), Fagerstrom Test for Cigarette Dependence (FTCD) score, nicotine metabolite ratio (NMR), number of days cigarettes were smoked within the last 30 days, and number of cigarettes smoked on a day with smoking for each participant (P).

|  | P1 | P2 | P3 | P4 | P5 | P6 | P7 | P8 | P9 | P10 | P11 | P12 | P13 | P14 | P15 |
| --- | --- | --- | --- | --- | --- | --- | --- | --- | --- | --- | --- | --- | --- | --- | --- |
| Age | 27 | 32 | 22 | 22 | 33 | 23 | 26 | 40 | 24 | 50 | 30 | 24 | 40 | 34 | 40 |
| Sex | f | f | m | F | m | m | m | f | f | f | f | f | m | m | m |
| Height | 170 | 168 | 175 | 165 | 174 | 178 | 185 | 166 | 165 | 174 | 174 | 162 | 173 | 176 | 172 |
| Weight | 82 | 85 | 62 | 64 | 116 | 92 | 88 | 85 | 68 | 64 | 60 | 48 | 73 | 88 | 96 |
| FTCD | 3 | 5 | 3 | 3 | 4 | 4 | 7 | 5 | 4 | 6 | 6 | 4 | 5 | 3 | 4 |
| NMR | 0.63 | 0.74 | 0.46 | 1.14 | 0.29 | 0.32 | 0.31 | 0.51 | 0.44 | 0.40 | 0.00 | 0.60 | 0.79 | 0.37 | 0.04 |
| Days smoked | 30 | 29 | 30 | 30 | 30 | 30 | 30 | 30 | 30 | 30 | 30 | 30 | 30 | 28 | 30 |
| Cigarettes smoked | 18 | 10 | 12 | 15 | 12 | 15 | 15 | 18 | 12 | 12 | 10 | 11 | 15 | 12 | 17 |

# 2. Individual plasma nicotine curves

Baseline-corrected individual plasma curves are presented in Supplementary Figure 1. In Supplementary Tables 2-6, the corresponding concentrations (prior to baseline-correction) are shown. Delays in blood sampling were considered. Remaining amounts of nicotine in the used pouches are presented in Supplementary Table 7. Unfortunately, one used pouch (20 mg) was not analyzed due to technical problems.

**
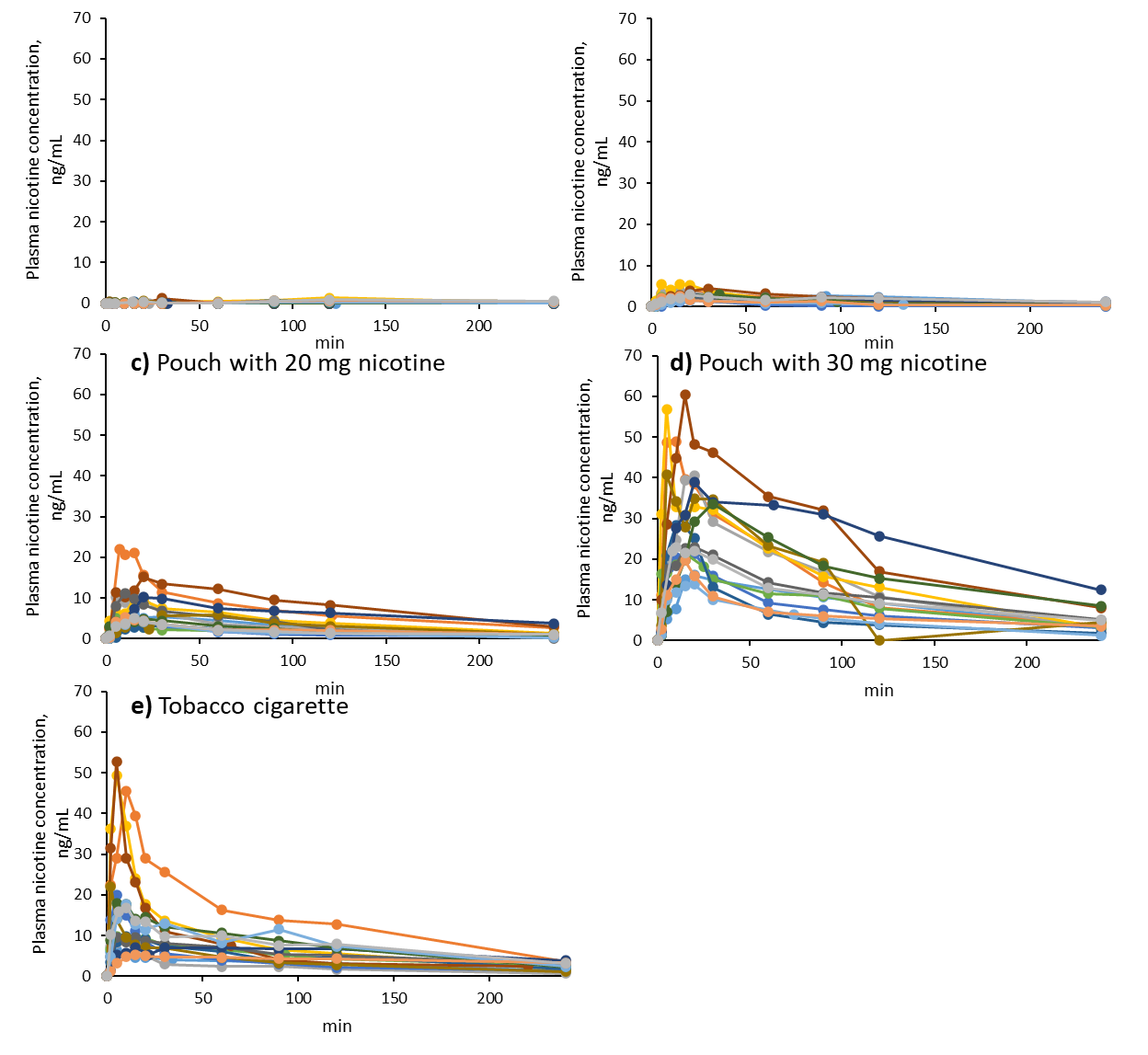
**
**Supplementary Figure 1.** Individual plasma curves per participant in the five study arms (a-e) after baseline correction.

**Supplementary Table 2.** Individual plasma nicotine concentrations (columns C) in ng/mL at the actual blood sampling time points (columns t) in min for each participant (P) in the nicotine-free pouch arm.

| P 1 | | P 2 | | P 3 | | P 4 | | P 5 | | P 6 | | P 7 | | P 8 | | P 9 | | P 10 | | P 11 | | P 12 | | P 13 | | P 14 | | P 15 | |
| --- | --- | --- | --- | --- | --- | --- | --- | --- | --- | --- | --- | --- | --- | --- | --- | --- | --- | --- | --- | --- | --- | --- | --- | --- | --- | --- | --- | --- | --- |
| t | C | t | C | t | C | t | C | t | C | t | C | T | C | T | C | t | C | t | C | t | C | t | C | t | C | t | C | t | C |
| 0 | 1.9 | 0 | 0.9 | 0 | 0.0 | 0 | 0.6 | 0 | 0.9 | 0 | 0.3 | 0 | 1.6 | 0 | 3.0 | 0 | 0.0 | 0 | 0.9 | 0 | 4.0 | 0 | 0.7 | 0 | 0.8 | 0 | 1.3 | 0 | 3.5 |
| 2 | 1.8 | 2 | 0.7 | 2 | 0.0 | 2 | 0.5 | 2 | 0.8 | 2 | 0.8 | 2 | 1.6 | 2 | 3.4 | 2 | 0.0 | 2 | 1.0 | 2 | 3.4 | 2 | 0.8 | 2 | 0.7 | 2 | 1.2 | 2 | 3.4 |
| 5 | 1.8 | 5 | 0.6 | 5 | 0.0 | 5 | 0.6 | 5 | 1.0 | 5 | 0.4 | 5 | 1.6 | 5 | 3.1 | 5 | 0.0 | 5 | 1.0 | 5 | 3.5 | 5 | 0.9 | 5 | 0.8 | 5 | 1.1 | 5 | 3.4 |
| 10 | 1.6 | 10 | 0.5 | 10 | 0.0 | 10 | 0.6 | 10 | 0.8 | 10 | 0.4 | 10 | 1.7 | 10 | 2.4 | 10 | 0.0 | 10 | 1.0 | 10 | 3.1 | 10 | 0.7 | 10 | 0.7 | 10 | 0.8 | 14 | 3.7 |
| 15 | 1.6 | 15 | 0.6 | 15 | 0.0 | 15 | 0.5 | 15 | 0.9 | 15 | 0.4 | 15 | 1.8 | 15 | 2.3 | 15 | 0.0 | 15 | 0.9 | 15 | 3.4 | 15 | 0.7 | 15 | 0.6 | 15 | 1.0 | - | - |
| 20 | 1.5 | 20 | 0.6 | 23 | 0.0 | 20 | 0.7 | 20 | 0.9 | 20 | 0.4 | 20 | 1.8 | 20 | 2.3 | 20 | 0.0 | 20 | 1.5 | 20 | 2.9 | 20 | 0.7 | 20 | 0.7 | 20 | 1.2 | 20 | 3.7 |
| 30 | 1.5 | 30 | 0.5 | 30 | 0.0 | 30 | 0.6 | 30 | 0.9 | 30 | 0.6 | 30 | 1.8 | 30 | 4.1 | 30 | 0.0 | 32 | 0.8 | 33 | 3.2 | 30 | 0.7 | 30 | 0.7 | 30 | 1.2 | 30 | 3.3 |
| 60 | 1.3 | 60 | 0.5 | 60 | 0.0 | 60 | 0.6 | 60 | 0.9 | 60 | 0.4 | 60 | 1.5 | 60 | 2.4 | 60 | 0.0 | 60 | 0.7 | 60 | 3.2 | 60 | 0.7 | 60 | 0.7 | 60 | 1.4 | 60 | 2.6 |
| 90 | 1.1 | 90 | 0.5 | 90 | 0.0 | 90 | 0.7 | 90 | 0.9 | 90 | 0.4 | 90 | 1.9 | 90 | 1.8 | 90 | 0.0 | 90 | 0.6 | 90 | 3.0 | 90 | 0.7 | 90 | 0.8 | 90 | 1.6 | 90 | 3.1 |
| 123 | 1.1 | 120 | 0.6 | 120 | 0.0 | 120 | 1.4 | 120 | 0.9 | 120 | 0.9 | 120 | 1.4 | 120 | 1.9 | 120 | 0.0 | 120 | 0.6 | 120 | 2.7 | 120 | 0.6 | 120 | 0.7 | 120 | 1.6 | 120 | 3.3 |
| 240 | 0.7 | 240 | 0.6 | 240 | 0.0 | 240 | 0.1 | 286 | 0.4 | 240 | 0.6 | 240 | 1.0 | 240 | 1.4 | 240 | 0.0 | 240 | 0.4 | 240 | 1.9 | 240 | 0.4 | 240 | 0.4 | 240 | 1.1 | 240 | 2.2 |

**Supplementary Table 3.** Individual plasma nicotine concentrations (columns C) in ng/mL at the actual blood sampling time points (columns t) in min for each participant (P) in the 6 mg nicotine pouch arm.

| P 1 | | P 2 | | P 3 | | P 4 | | P 5 | | P 6 | | P 7 | | P 8 | | P 9 | | P 10 | | P 11 | | P 12 | | P 13 | | P 14 | | P 15 | |
| --- | --- | --- | --- | --- | --- | --- | --- | --- | --- | --- | --- | --- | --- | --- | --- | --- | --- | --- | --- | --- | --- | --- | --- | --- | --- | --- | --- | --- | --- |
| t | C | t | C | t | C | T | C | t | C | t | C | T | C | T | C | t | C | t | C | t | C | t | C | t | C | t | C | t | C |
| 0 | 2.1 | 0 | 0.5 | 0 | 0.0 | 0 | 0.5 | 0 | 1.7 | 0 | 0.5 | 0 | 1.9 | 0 | 0.8 | 0 | 0.2 | 0 | 1.8 | 0 | 1.4 | 0 | 0.6 | 0 | 0.3 | 0 | 1.4 | 0 | 4.3 |
| 2 | 2.7 | 2 | 0.8 | 2 | 0.3 | 2 | 1.9 | - | - | 2 | 1.0 | 2 | 1.8 | 2 | 1.2 | 2 | 0.2 | 2 | 1.6 | 2 | 1.6 | 2 | 0.8 | 2 | 0.4 | 2 | 2.0 | 2 | 5.1 |
| 5 | 2.6 | 5 | 3.6 | 5 | 3.3 | 5 | 5.8 | 5 | 1.8 | 5 | 2.9 | 5 | 3.1 | 5 | 2.2 | 5 | 1.2 | 5 | 2.6 | 5 | 2.1 | 5 | 1.3 | 5 | 0.7 | 5 | 3.4 | 5 | 5.3 |
| 10 | 3.3 | 10 | 2.8 | 10 | 3.7 | 10 | 4.6 | 10 | 2.8 | 10 | 2.9 | 10 | 3.7 | 10 | 3.3 | 10 | 2.0 | 10 | 3.3 | 10 | 2.8 | 10 | 2.0 | 10 | 1.2 | 10 | 3.5 | 10 | 5.8 |
| 15 | 4.4 | 15 | 3.3 | 15 | 3.8 | 15 | 5.8 | 15 | 2.9 | 15 | 3.1 | 15 | 4.0 | 15 | 3.4 | 15 | 2.5 | 15 | 3.4 | 15 | 3.6 | 15 | 2.3 | 15 | 1.6 | 15 | 3.7 | 15 | 6.1 |
| 20 | 4.5 | 20 | 4.1 | 20 | 3.5 | 20 | 5.6 | 20 | 3.4 | 20 | 3.2 | 20 | 4.0 | 20 | 4.5 | 20 | 2.0 | 20 | 4.3 | 20 | 4.4 | 20 | 3.1 | 20 | 1.7 | 20 | 2.9 | 20 | 6.8 |
| 30 | 3.5 | 30 | 3.6 | 30 | 2.9 | 30 | 4.1 | 30 | 2.8 | 30 | 2.7 | 30 | 3.8 | 30 | 5.0 | 30 | 2.0 | 30 | 4.0 | 30 | 3.6 | 36 | 3.5 | 30 | 1.6 | 30 | 2.6 | 30 | 6.1 |
| 60 | 3.5 | 60 | 2.7 | 60 | 1.4 | 60 | 2.3 | 60 | 1.8 | 60 | 1.9 | 60 | 2.6 | 60 | 3.6 | 60 | 2.3 | 60 | 3.3 | 60 | 2.7 | 60 | 2.4 | 60 | 1.0 | 60 | 2.2 | 60 | 4.8 |
| 92 | 3.5 | 90 | 2.0 | 90 | 1.2 | 90 | 2.0 | 90 | 1.5 | 90 | 1.6 | 90 | 2.5 | 90 | 2.8 | 90 | 1.6 | 90 | 2.7 | 90 | 3.4 | 98 | 1.6 | 90 | 1.1 | 90 | 2.4 | 90 | 5.1 |
| 120 | 3.0 | 122 | 1.4 | 120 | 0.9 | 120 | 1.3 | 120 | 1.2 | 120 | 1.9 | 120 | 2.4 | 120 | 2.3 | 120 | 1.3 | 120 | 2.1 | 120 | 2.5 | 120 | 1.0 | 133 | 0.6 | 120 | 1.5 | 120 | 4.4 |
| 240 | 1.0 | 240 | 0.6 | 240 | 0.0 | 240 | 0.3 | 240 | 0.9 | 240 | 0.9 | 240 | 1.0 | 240 | 1.1 | 240 | 0.6 | 240 | 1.0 | 240 | 1.8 | 240 | 0.6 | 240 | 0.4 | 240 | 1.1 | 240 | 2.5 |

**Supplementary Table 4.** Individual plasma nicotine concentrations (columns C) in ng/mL at the actual blood sampling time points (columns t) in min for each participant (P) in the 20 mg nicotine pouch arm.

| P 1 | | P 2 | | P 3 | | P 4 | | P 5 | | P 6 | | P 7 | | P 8 | | P 9 | | P 10 | | P 11 | | P 12 | | P 13 | | P 14 | | P 15 | |
| --- | --- | --- | --- | --- | --- | --- | --- | --- | --- | --- | --- | --- | --- | --- | --- | --- | --- | --- | --- | --- | --- | --- | --- | --- | --- | --- | --- | --- | --- |
| t | C | t | C | t | C | t | C | t | C | t | C | T | C | T | C | t | C | t | C | t | C | t | C | t | C | t | C | t | C |
| 0 | 0.7 | 0 | 0.3 | 0 | 0.4 | 0 | 0.8 | 0 | 0.6 | 0 | 0.3 | 0 | 2.0 | 0 | 3.4 | 0 | 0.4 | 0 | 0.8 | 0 | 1.7 | 0 | 0.9 | 0 | 0.5 | 0 | 1.4 | 0 | 2.6 |
| 2 | 0.6 | 2 | 2.7 | 2 | 1.4 | 2 | 5.1 | 2 | 0.7 | 2 | 1.4 | 2 | 2.0 | 2 | 6.1 | 2 | 1.3 | 2 | 0.7 | 2 | 2.2 | 2 | 3.9 | 2 | 0.6 | 2 | 1.7 | 2 | 3.3 |
| 5 | 2.4 | 7 | 22.3 | 5 | 10.6 | 5 | 7.3 | 5 | 2.6 | 5 | 5.4 | 5 | 2.2 | 5 | 14.6 | 5 | 8.3 | 5 | 2.1 | 5 | 5.0 | 5 | 5.0 | 5 | 3.9 | 5 | 5.5 | 5 | 5.5 |
| 10 | 4.1 | 10 | 21.0 | 10 | 9.2 | 10 | 6.8 | 10 | 4.0 | 10 | 4.4 | 10 | 4.2 | 10 | 13.3 | 10 | 11.3 | 10 | 3.4 | 10 | 6.7 | 10 | 4.8 | 10 | 3.9 | 10 | 6.6 | 10 | 6.3 |
| 15 | 6.8 | 15 | 21.5 | 15 | 11.0 | 15 | 10.3 | 15 | 4.3 | 15 | 3.5 | 15 | 4.6 | 15 | 14.9 | 15 | 10.0 | 15 | 5.0 | 15 | 8.8 | 15 | 5.1 | 15 | 5.2 | 15 | 5.9 | 15 | 7.6 |
| 20 | 5.7 | 20 | 16.1 | 20 | 10.6 | 20 | 10.2 | 20 | 4.3 | 20 | 3.0 | 20 | 4.7 | 20 | 18.1 | 20 | 8.5 | 23 | 3.0 | 20 | 11.7 | 20 | 5.7 | 20 | 4.5 | 20 | 5.7 | 20 | 6.8 |
| 30 | 5.8 | 30 | 11.7 | 30 | 6.3 | 30 | 8.1 | 30 | 3.3 | 30 | 2.5 | 30 | 5.1 | 30 | 16.2 | 30 | 6.8 | 30 | 6.3 | 30 | 11.4 | 30 | 5.3 | 30 | 3.7 | 30 | 4.8 | 30 | 6.0 |
| 60 | 4.8 | 60 | 9.0 | 60 | 3.9 | 60 | 6.9 | 60 | 2.2 | 60 | 2.3 | 60 | 4.0 | 60 | 14.3 | 60 | 5.5 | 60 | 6.3 | 60 | 8.8 | 60 | 3.7 | 60 | 2.0 | 60 | 3.2 | 60 | 4.6 |
| 90 | 3.7 | 90 | 7.1 | 90 | 3.1 | 90 | 4.9 | 90 | 1.6 | 90 | 2.1 | 90 | 3.1 | 90 | 11.1 | 90 | 4.3 | 90 | 4.2 | 90 | 7.9 | 90 | 3.0 | 90 | 1.6 | 90 | 3.0 | 90 | 4.0 |
| 120 | 2.9 | 121 | 5.8 | 120 | 2.7 | 120 | 4.2 | 120 | 1.3 | 120 | 1.7 | 120 | 2.9 | 120 | 9.5 | 120 | 2.1 | 120 | 3.4 | 120 | 7.3 | 120 | 2.1 | 120 | 1.4 | 120 | 2.9 | 120 | 3.5 |
| 240 | 0.8 | 240 | 2.8 | 240 | 1.1 | 240 | 1.5 | 245 | 0.8 | 240 | 1.0 | 240 | 1.8 | 240 | 3.4 | - | - | 240 | 1.3 | 240 | 4.3 | 240 | 1.0 | 240 | 0.2 | 240 | 1.5 | 240 | 2.8 |

**Supplementary Table 5.** Individual plasma nicotine concentrations (columns C) in ng/mL at the actual blood sampling time points (columns t) in min for each participant (P) in the 30 mg nicotine pouch arm.

| P 1 | | P 2 | | P 3 | | P 4 | | P 5 | | P 6 | | P 7 | | P 8 | | P 9 | | P 10 | | P 11 | | P 12 | | P 13 | | P 14 | | P 15 | |
| --- | --- | --- | --- | --- | --- | --- | --- | --- | --- | --- | --- | --- | --- | --- | --- | --- | --- | --- | --- | --- | --- | --- | --- | --- | --- | --- | --- | --- | --- |
| T | C | T | C | t | C | T | C | t | C | t | C | T | C | T | C | t | C | t | C | t | C | t | C | t | C | t | C | t | C |
| 0 | 0.5 | 0 | 0.3 | 0 | 0.3 | 0 | 0.2 | 0 | 1.5 | 0 | 0.4 | 0 | 0.9 | 0 | 1.9 | 0 | 0.2 | 0 | 1.3 | 0 | 2.0 | 0 | 1.0 | 0 | 0.2 | 0 | 2.2 | 0 | 2.0 |
| 2 | 1.9 | 2 | 11.4 | 2 | 6.8 | 2 | 31.2 | 2 | 3.6 | 2 | 16.8 | 2 | 2.4 | 2 | 12.0 | 2 | 2.4 | 2 | 5.1 | 2 | 3.9 | 2 | 2.2 | 2 | 1.7 | 2 | 5.0 | 2 | 8.9 |
| 5 | 5.7 | 5 | 48.9 | 5 | 17.9 | 5 | 57.1 | 6 | 19.1 | 5 | 17.0 | 5 | 21.8 | 5 | 30.4 | 5 | 17.6 | 5 | 42.2 | 5 | 15.6 | 5 | 8.1 | 5 | 10.2 | 5 | 13.4 | 8 | 24.0 |
| 10 | 8.3 | 10 | 49.1 | 10 | 25.0 | 10 | 33.0 | 10 | 21.8 | 10 | 18.9 | 10 | 29.1 | 10 | 46.6 | 10 | 18.6 | 10 | 35.5 | 10 | 29.6 | 10 | 14.9 | 10 | 12.0 | 10 | 17.1 | 10 | 24.8 |
| 15 | 15.4 | 15 | 40.0 | 15 | 39.7 | 15 | 31.0 | 15 | 22.1 | 15 | 21.7 | 15 | 28.8 | 15 | 62.1 | 15 | 22.8 | 15 | 29.1 | 15 | 32.7 | 15 | 20.7 | 15 | 13.6 | 15 | 21.8 | 15 | 23.4 |
| 20 | 16.5 | 20 | 38.7 | 20 | 40.7 | 20 | 33.0 | 20 | 22.7 | 25 | 18.5 | 20 | 25.9 | 20 | 49.8 | 20 | 23.2 | 20 | 36.2 | 20 | 40.6 | 20 | 30.1 | 20 | 14.0 | 20 | 18.0 | 20 | 23.9 |
| 30 | 15.3 | 30 | 31.5 | 30 | 29.4 | 30 | 32.3 | 30 | 17.2 | 30 | 15.3 | 30 | 13.9 | 30 | 47.8 | 30 | 21.2 | 30 | 36.0 | 30 | 35.7 | 30 | 34.4 | 30 | 10.2 | 30 | 12.9 | 30 | 21.7 |
| 60 | 12.9 | 60 | 23.2 | 60 | 22.0 | 60 | 22.7 | 60 | 10.4 | 60 | 11.8 | 60 | 7.1 | 60 | 36.7 | 60 | 14.4 | 60 | 24.6 | 63 | 34.6 | 60 | 26.1 | 74 | 6.4 | 60 | 8.8 | 60 | 14.4 |
| 90 | 10.9 | 90 | 14.4 | 90 | 17.0 | 90 | 15.8 | 90 | 8.5 | 90 | 11.2 | 90 | 5.0 | 90 | 33.1 | 90 | 11.8 | 90 | 20.4 | 90 | 32.2 | 90 | 18.9 | 90 | 5.5 | 90 | 7.6 | 90 | 12.6 |
| 120 | 9.4 | 120 | 8.3 | 120 | 11.0 | 120 | 13.2 | 120 | 6.9 | 120 | 8.1 | 120 | 4.3 | 120 | 17.9 | 120 | 10.6 | - | - | 120 | 26.7 | 120 | 15.8 | 120 | 4.2 | 120 | 6.9 | 120 | 10.3 |
| 240 | 3.4 | 240 | 4.0 | 240 | 3.1 | 240 | 3.1 | 240 | 3.5 | 240 | 3.4 | 240 | 2.1 | 240 | 8.4 | 240 | 5.2 | 240 | 5.9 | 240 | 13.0 | 240 | 8.8 | 240 | 1.3 | 240 | 4.4 | 240 | 5.6 |

**Supplementary Table 6.** Individual plasma nicotine concentrations (columns C) in ng/mL at the actual blood sampling time points (columns t) in min for each participant (P) in the tobacco cigarette arm.

| P 1 | | P 2 | | P 3 | | P 4 | | P 5 | | P 6 | | P 7 | | P 8 | | P 9 | | P 10 | | P 11 | | P 12 | | P 13 | | P 14 | | P 15 | |
| --- | --- | --- | --- | --- | --- | --- | --- | --- | --- | --- | --- | --- | --- | --- | --- | --- | --- | --- | --- | --- | --- | --- | --- | --- | --- | --- | --- | --- | --- |
| t | C | t | C | t | C | T | C | t | C | t | C | T | C | T | C | t | C | t | C | t | C | t | C | t | C | t | C | t | C |
| 0 | 1.0 | 0 | 0.4 | 0 | 0.3 | 0 | 0.0 | 0 | 1.1 | 0 | 0.6 | 0 | 2.1 | 0 | 1.8 | 0 | 0.1 | 0 | 0.6 | 0 | 1.9 | 0 | 0.2 | 0 | 0.5 | 0 | 2.0 | 0 | 2.7 |
| 2 | 6.9 | 2 | 22.9 | 2 | 7.5 | 2 | 36.2 | 2 | 14.9 | 2 | 7.3 | 2 | 5.6 | 2 | 33.2 | 2 | 4.9 | 2 | 22.6 | 2 | 3.4 | 2 | 9.0 | 2 | 5.4 | 2 | 3.3 | 2 | 13.0 |
| 5 | 7.4 | 5 | 29.5 | 5 | 8.5 | 5 | 49.4 | 5 | 21.1 | 5 | 10.3 | 5 | 10.5 | 5 | 54.6 | 5 | 9.7 | 5 | 14.6 | 5 | 7.1 | 5 | 18.1 | 5 | 14.5 | 5 | 5.3 | 6 | 18.5 |
| 10 | 6.4 | 10 | 46.0 | 10 | 8.7 | 10 | 36.8 | 10 | 16.0 | 10 | 10.1 | 10 | 11.7 | 10 | 30.7 | 10 | 8.6 | 10 | 10.1 | 10 | 7.6 | 10 | 17.8 | 10 | 18.2 | 10 | 6.8 | 10 | 19.3 |
| 15 | 5.5 | 15 | 39.8 | 15 | 5.8 | 15 | 24.1 | 15 | 12.1 | 15 | 9.9 | 15 | 11.1 | 15 | 24.9 | 15 | 9.7 | 15 | 8.3 | 15 | 8.9 | 15 | 14.2 | 15 | 13.1 | 15 | 7.2 | 15 | 16.0 |
| 20 | 5.5 | 20 | 29.3 | 20 | 5.5 | 20 | 17.7 | 20 | 9.6 | 20 | 10.0 | 20 | 10.8 | 20 | 18.5 | 20 | 8.9 | 20 | 7.6 | 22 | 7.0 | 20 | 14.7 | 20 | 11.8 | 20 | 7.0 | 20 | 15.9 |
| 34 | 4.8 | 30 | 26.0 | 30 | 3.2 | 30 | 13.7 | 30 | 6.5 | 30 | 8.2 | 30 | 9.1 | 30 | 12.7 | 30 | 8.1 | 30 | 7.5 | 30 | 8.7 | 30 | 12.2 | 30 | 13.4 | 30 | 6.7 | 30 | 11.9 |
| 60 | 4.5 | 60 | 16.5 | 60 | 2.6 | 60 | 9.4 | 60 | 4.8 | 60 | 7.1 | 60 | 7.3 | 65 | 9.1 | 60 | 7.2 | 60 | 5.1 | 60 | 8.5 | 60 | 10.8 | 60 | 8.6 | 60 | 6.5 | 60 | 11.7 |
| 102 | 3.8 | 90 | 14.0 | 90 | 2.6 | 90 | 6.6 | 90 | 3.9 | 93 | 5.7 | 90 | 4.7 | 90 | 5.6 | 90 | 5.6 | 90 | 3.6 | 90 | 8.0 | 90 | 8.8 | 90 | 11.7 | 90 | 6.0 | 90 | 9.0 |
| 120 | 3.6 | 120 | 12.9 | 120 | 1.8 | 120 | 5.7 | 120 | 2.9 | 120 | 4.6 | 120 | 5.8 | 120 | 4.5 | 120 | 5.3 | 120 | 3.1 | 120 | 7.9 | 120 | 7.0 | 120 | 7.6 | 120 | 5.8 | 120 | 9.1 |
| 240 | 1.4 | 240 | 3.6 | 240 | 0.8 | 240 | 1.6 | 242 | 1.8 | 240 | 2.8 | 240 | 2.2 | 220 | 3.7 | 240 | 3.0 | 240 | 1.3 | 240 | 4.5 | 240 | 2.3 | 240 | 2.4 | 240 | 4.5 | 240 | 3.7 |

**Supplementary Table 7.** Remaining nicotine contents in used pouches.

| Nominal total amount | Analyzed total amount, mg | Remaining nicotine amount after use, mg | | | | | | | | | | | | | | |
| --- | --- | --- | --- | --- | --- | --- | --- | --- | --- | --- | --- | --- | --- | --- | --- | --- |
|  |  | P 1 | P 2 | P 3 | P 4 | P 5 | P 6 | P 7 | P 8 | P 9 | P 10 | P 11 | P 12 | P 13 | P 14 | P 15 |
| 6 mg | 4.8 ± 0.4 | 3.1 | 3.5 | 4.6 | 3.9 | 3.7 | 2.1 | 2.2 | 2.4 | 3.6 | 1.9 | 2.3 | 2.1 | 3.0 | 3.0 | 3.1 |
| 20 mg | 16.3 ± 3.1 | 12.8 | 14.6 | 10.7 | 14.5 | 12.7 | 12.9 | 11.5 | 11.6 | 14.5 | N/A | 10.9 | 11.2 | 12.5 | 11.8 | 11.1 |
| 30 mg | 27.1 ± 0.2 | 17.3 | 17.4 | 14.3 | 14.4 | 11.9 | 16.8 | 11.3 | 9.2 | 16.8 | 13.4 | 11.0 | 11.5 | 10.8 | 9.6 | 9.7 |

N/A: Sample missing

# 3. Craving

Acute craving for a cigarette was inquired at several time points with one question (“I now feel the urge for a cigarette”) answered on a scale from 1 (lowest) to 7 (highest). Individual craving scores per time point are presented in Supplementary Tables 8-12.

All products have led to a significant reduction of craving: nicotine-free pouch (p = 0.0004), 6 mg nicotine pouch (p = 0.02), 20 mg nicotine pouch (p = 0.000002), 30 mg nicotine pouch (p = 0.00005), and tobacco cigarette (p = 0.00004).

**Supplementary Table 8.** Individual craving scores (“I now feel the urge for a cigarette” on a scale from 1 (lowest) to 7 (highest)) for each participant (P) in the nicotine-free pouch arm.

|  | P 1 | P 2 | P 3 | P 4 | P 5 | P 6 | P 7 | P 8 | P 9 | P 10 | P 11 | P 12 | P 13 | P 14 | P 15 |
| --- | --- | --- | --- | --- | --- | --- | --- | --- | --- | --- | --- | --- | --- | --- | --- |
| Baseline | 6 | 7 | 1 | 4 | 5 | 1 | 7 | 3 | 7 | 6 | 4 | 7 | 7 | 2 | 4 |
| 2 min | 6 | 5 | 1 | 1 | 5 | 1 | 7 | 1 | 7 | 6 | 4 | 7 | 4 | 1 | 3 |
| 5 min | 6 | 5 | 1 | 1 | 3 | 1 | 4 | 1 | 7 | 4 | 4 | 4 | 3 | 1 | 3 |
| 10 min | 6 | 4 | 1 | 1 | 1 | 1 | 5 | 1 | 6 | 4 | 4 | 2 | 2 | 1 | 3 |
| 15 min | 5 | 5 | 1 | 1 | 1 | 1 | 6 | 1 | 5 | 4 | 4 | 2 | 1 | 1 | 4 |
| 20 min | 6 | 5 | 1 | 1 | 1 | 1 | 5 | 1 | 5 | 4 | 4 | 2 | 1 | 1 | 4 |
| 30 min | 5 | 6 | 1 | 1 | 3 | 4 | 7 | 1 | 5 | 4 | 3 | 2 | 1 | 1 | 2 |
| 60 min | 5 | 6 | 1 | 1 | 5 | 5 | 7 | 1 | 5 | 5 | 4 | 4 | 1 | 1 | 4 |
| 90 min | 5 | 7 | 1 | 1 | 1 | 5 | 7 | 1 | 5 | 5 | 4 | 4 | 1 | 1 | 4 |
| 120 min | 5 | 7 | 1 | 3 | 3 | 5 | 7 | 1 | 5 | 6 | 5 | 4 | 1 | 1 | 4 |
| 240 min | 6 | 7 | 5 | 4 | 7 | 3 | 7 | 2 | 5 | 7 | 6 | 5 | 1 | 2 | 5 |

**Supplementary Table 9.** Individual craving scores (“I now feel the urge for a cigarette” on a scale from 1 (lowest) to 7 (highest)) for each participant (P) in the 6 mg nicotine pouch arm.

|  | P 1 | P 2 | P 3 | P 4 | P 5 | P 6 | P 7 | P 8 | P 9 | P 10 | P 11 | P 12 | P 13 | P 14 | P 15 |
| --- | --- | --- | --- | --- | --- | --- | --- | --- | --- | --- | --- | --- | --- | --- | --- |
| Baseline | 5 | 7 | 2 | 5 | 3 | 1 | 7 | 1 | 6 | 7 | 2 | 2 | 7 | 3 | 7 |
| 2 min | 5 | 7 | 1 | 1 | 1 | 2 | 7 | 1 | 6 | 5 | 3 | 1 | 3 | 2 | 6 |
| 5 min | 4 | 7 | 1 | 1 | 1 | 3 | 5 | 1 | 6 | 3 | 3 | 1 | 3 | 2 | 5 |
| 10 min | 4 | 7 | 1 | 1 | 1 | 3 | 6 | 1 | 4 | 1 | 3 | 1 | 1 | 1 | 5 |
| 15 min | 4 | 7 | 1 | 1 | 1 | 3 | 7 | 1 | 4 | 3 | 3 | 1 | 1 | 1 | 4 |
| 20 min | 4 | 7 | 1 | 1 | 1 | 2 | 7 | 1 | 3 | 4 | 3 | 3 | 1 | 1 | 3 |
| 30 min | 4 | 7 | 1 | 1 | 1 | 3 | 7 | 1 | 4 | 4 | 3 | 7 | 1 | 1 | 2 |
| 60 min | 3 | 7 | 1 | 2 | 1 | 1 | 7 | 1 | 4 | 6 | 3 | 7 | 2 | 2 | 1 |
| 90 min | 4 | 7 | 2 | 2 | 1 | 3 | 7 | 1 | 4 | 6 | 4 | 7 | 2 | 1 | 3 |
| 120 min | 3 | 7 | 2 | 3 | 3 | 5 | 7 | 1 | 4 | 7 | 4 | 7 | 2 | 1 | 2 |
| 240 min | 5 | 7 | 4 | 3 | 4 | 5 | 7 | 1 | 5 | 7 | 5 | 7 | 2 | 3 | 4 |

**Supplementary Table 10.** Individual craving scores (“I now feel the urge for a cigarette” on a scale from 1 (lowest) to 7 (highest)) for each participant (P) in the 20 mg nicotine pouch arm.

|  | P 1 | P 2 | P 3 | P 4 | P 5 | P 6 | P 7 | P 8 | P 9 | P 10 | P 11 | P 12 | P 13 | P 14 | P 15 |
| --- | --- | --- | --- | --- | --- | --- | --- | --- | --- | --- | --- | --- | --- | --- | --- |
| Baseline | 5 | 7 | 1 | 2 | 7 | 5 | 7 | 1 | 6 | 7 | 5 | 7 | 5 | 3 | 5 |
| 2 min | 4 | 7 | 1 | 1 | 1 | 5 | 6 | 1 | 6 | 5 | 5 | 1 | 3 | 2 | 5 |
| 5 min | 3 | 5 | 1 | 1 | 1 | 5 | 5 | 1 | 5 | 3 | 5 | 1 | 1 | 1 | 4 |
| 10 min | 4 | 4 | 1 | 1 | 1 | 4 | 6 | 1 | 4 | 1 | 4 | 1 | 1 | 1 | 2 |
| 15 min | 4 | 3 | 1 | 1 | 1 | 3 | 7 | 1 | 4 | 1 | 4 | 1 | 1 | 1 | 2 |
| 20 min | 4 | 2 | 1 | 1 | 1 | 4 | 7 | 1 | 3 | 1 | 4 | 1 | 1 | 1 | 1 |
| 30 min | 4 | 3 | 1 | 1 | 2 | 5 | 7 | 1 | 3 | 1 | 3 | 1 | 1 | 1 | 1 |
| 60 min | 5 | 4 | 2 | 1 | 3 | 5 | 7 | 1 | 4 | 3 | 4 | 3 | 1 | 1 | 1 |
| 90 min | 5 | 5 | 2 | 2 | 1 | 6 | 7 | 1 | 4 | 4 | 4 | 4 | 1 | 1 | 1 |
| 120 min | 5 | 6 | 3 | 3 | 3 | 6 | 7 | 1 | 4 | 5 | 4 | 4 | 1 | 3 | 2 |
| 240 min | 6 | 7 | 4 | 3 | 6 | 6 | 7 | 2 | 5 | 7 | 5 | 4 | 1 | 3 | 3 |

**Supplementary Table 11.** Individual craving scores (“I now feel the urge for a cigarette” on a scale from 1 (lowest) to 7 (highest)) for each participant (P) in the 30 mg nicotine pouch arm.

|  | P 1 | P 2 | P 3 | P 4 | P 5 | P 6 | P 7 | P 8 | P 9 | P 10 | P 11 | P 12 | P 13 | P 14 | P 15 |
| --- | --- | --- | --- | --- | --- | --- | --- | --- | --- | --- | --- | --- | --- | --- | --- |
| Baseline | 6 | 7 | 1 | 5 | 2 | 7 | 7 | 1 | 6 | 6 | 5 | 4 | 7 | 2 | 5 |
| 2 min | 5 | 6 | 1 | 3 | 1 | 5 | 6 | 1 | 5 | 1 | 4 | 1 | 5 | 2 | 5 |
| 5 min | 2 | 3 | 1 | 1 | 1 | 2 | 2 | 1 | 4 | 3 | 3 | 1 | 5 | 1 | 5 |
| 10 min | 3 | 2 | 1 | 1 | 2 | 1 | 1 | 1 | 3 | 1 | 3 | 1 | 3 | 1 | 4 |
| 15 min | 4 | 3 | 1 | 1 | 2 | 1 | 1 | 1 | 3 | 1 | 2 | 1 | 2 | 1 | 4 |
| 20 min | 3 | 3 | 1 | 1 | 1 | 1 | 1 | 1 | 3 | 1 | 2 | 1 | 1 | 1 | 4 |
| 30 min | 3 | 4 | 1 | 2 | 2 | 2 | 1 | 1 | 3 | 3 | 2 | 1 | 1 | 1 | 1 |
| 60 min | 4 | 5 | 1 | 3 | 1 | 4 | 3 | 1 | 3 | 5 | 2 | 1 | 1 | 1 | 1 |
| 90 min | 5 | 6 | 2 | 3 | 2 | 5 | 5 | 1 | 3 | 6 | 2 | 1 | 1 | 1 | 1 |
| 120 min | 5 | 7 | 2 | 4 | 4 | 7 | 7 | 1 | 3 | 6 | 4 | 1 | 2 | 1 | 3 |
| 240 min | 6 | 7 | 4 | 6 | 6 | 6 | 7 | 1 | 3 | 7 | 5 | 1 | 3 | 2 | 3 |

**Supplementary Table 12.** Individual craving scores (“I now feel the urge for a cigarette” on a scale from 1 (lowest) to 7 (highest)) for each participant (P) in the tobacco cigarette arm.

|  | P 1 | P 2 | P 3 | P 4 | P 5 | P 6 | P 7 | P 8 | P 9 | P 10 | P 11 | P 12 | P 13 | P 14 | P 15 |
| --- | --- | --- | --- | --- | --- | --- | --- | --- | --- | --- | --- | --- | --- | --- | --- |
| Baseline | 6 | 7 | 1 | 3 | 3 | 1 | 7 | 3 | 5 | 7 | 3 | 7 | 6 | 4 | 5 |
| 2 min | 1 | 5 | 1 | 6 | 1 | 7 | 4 | 1 | 7 | 2 | 1 | 1 | 4 | 1 | 4 |
| 5 min | 1 | 5 | 1 | 1 | 1 | 1 | 1 | 1 | 5 | 1 | 1 | 1 | 7 | 1 | 1 |
| 10 min | 2 | 2 | 1 | 1 | 1 | 2 | 1 | 1 | 2 | 1 | 1 | 1 | 4 | 1 | 1 |
| 15 min | 2 | 1 | 1 | 1 | 1 | 5 | 2 | 1 | 3 | 1 | 1 | 1 | 4 | 1 | 1 |
| 20 min | 2 | 1 | 1 | 1 | 1 | 5 | 5 | 1 | 2 | 5 | 1 | 1 | 7 | 1 | 1 |
| 30 min | 2 | 1 | 1 | 1 | 1 | 5 | 4 | 1 | 2 | 5 | 1 | 1 | 6 | 1 | 1 |
| 60 min | 3 | 5 | 1 | 1 | 1 | 1 | 7 | 1 | 3 | 5 | 3 | 5 | 7 | 1 | 3 |
| 90 min | 3 | 6 | 1 | 1 | 1 | 7 | 7 | 2 | 4 | 6 | 3 | 7 | 5 | 1 | 5 |
| 120 min | 4 | 6 | 1 | 1 | 1 | 1 | 7 | 2 | 4 | 6 | 4 | 7 | 7 | 1 | 5 |
| 240 min | 6 | 7 | 2 | 2 | 2 | 7 | 7 | 3 | 4 | 7 | 5 | 7 | 7 | 2 | 6 |

# 4. Cardiovascular effects and arterial stiffness

Mean results for heart rate and systolic and diastolic blood pressure are shown in Supplementary Tables 13-15.

**Supplementary Table 13.** Heart rate in beats per minute (bpm) measured at eight time points as mean and standard deviation (SD) for the five study arms.

|  | Nicotine-free pouches | | | 6 mg nicotine pouches | | | 20 mg nicotine pouches | | | 30 mg nicotine pouches | | | Tobacco cigarette | | |
| --- | --- | --- | --- | --- | --- | --- | --- | --- | --- | --- | --- | --- | --- | --- | --- |
|  | Mean | SD | p-Value | Mean | SD | p-value | Mean | SD | p-value | Mean | SD | p-value | Mean | SD | p-value |
| Baseline | 66.1 | 11.9 |  | 72.2 | 8.4 |  | 72.5 | 10.1 |  | 71.7 | 9.0 |  | 72.8 | 11.1 |  |
| 5 min | 70.7 | 12.8 | .722 | 77.8 | 8.0 | .593 | 86.1 | 13.7 | .668 | 96.5 | 14.1 | .108 | 94.6 | 13.4 | .333 |
| 20 min | 68.9 | 12.2 | .572 | 77.1 | 7.8 | .132 | 82.1 | 11.1 | .390 | 92.0 | 12.1 | .002 | 79.7 | 10.7 | .078 |
| 40 min | 68.6 | 12.3 | .564 | 75.2 | 7.9 | .749 | 77.4 | 10.5 | .490 | 86.3 | 12.6 | .868 | 79.2 | 9.2 | .411 |
| 60 min | 65.5 | 13.2 | .191 | 71.3 | 7.5 | .222 | 73.9 | 9.2 | .978 | 75.5 | 10.8 | .837 | 73.9 | 10.9 | .127 |
| 90 min | 64.1 | 12.6 | .180 | 67.7 | 6.5 | .218 | 71.7 | 9.8 | .761 | 75.5 | 11.9 | .641 | 71.9 | 13.1 | 1.000 |
| 120 min | 64.8 | 11.1 | .825 | 66.1 | 7.9 | .665 | 69.9 | 7.7 | .175 | 70.7 | 12.0 | .850 | 70.2 | 12.1 | .402 |
| 240 min | 71.9 | 12.5 | .123 | 71.3 | 8.7 | .036 | 75.3 | 14.3 | .215 | 77.5 | 11.5 | .982 | 71.3 | 12.2 | .101 |

**Supplementary Table 14.** Systolic blood pressure in mm Hg measured at eight time points as mean and standard deviation (SD) for the five study arms.

|  | Nicotine-free pouches | | | 6 mg nicotine pouches | | | 20 mg nicotine pouches | | | 30 mg nicotine pouches | | | Tobacco cigarette | | |
| --- | --- | --- | --- | --- | --- | --- | --- | --- | --- | --- | --- | --- | --- | --- | --- |
|  | Mean | SD | p-Value | Mean | SD | p-value | Mean | SD | p-value | Mean | SD | p-value | Mean | SD | p-value |
| Baseline | 115.1 | 12.9 |  | 116.6 | 15.5 |  | 116.9 | 16.7 |  | 115.4 | 15.0 |  | 114.7 | 12.2 |  |
| 5 min | 118.7 | 14.1 | .068 | 123.0 | 17.0 | .000 | 126.9 | 15.7 | .000 | 125.7 | 13.5 | .000 | 127.0 | 15.1 | .000 |
| 20 min | 118.1 | 15.3 | .240 | 126.2 | 15.7 | .000 | 124.1 | 14.7 | .003 | 130.3 | 14.0 | .000 | 125.9 | 12.7 | .000 |
| 40 min | 116.9 | 14.2 | .459 | 118.9 | 18.6 | .348 | 120.8 | 14.1 | .171 | 121.5 | 12.9 | .209 | 121.2 | 14.1 | .199 |
| 60 min | 121.4 | 18.6 | .360 | 121.5 | 14.8 | .391 | 119.6 | 13.5 | .646 | 121.9 | 13.1 | .209 | 123.3 | 20.6 | .209 |
| 90 min | 122.1 | 20.3 | .296 | 121.7 | 16.7 | .353 | 119.3 | 13.1 | .644 | 123.5 | 12.6 | .117 | 116.8 | 15.2 | .715 |
| 120 min | 117.5 | 17.3 | .708 | 117.8 | 15.2 | .837 | 123.7 | 19.2 | .322 | 121.0 | 15.9 | .344 | 119.9 | 17.5 | .435 |
| 240 min | 122.3 | 17.7 | .250 | 124.4 | 16.8 | .188 | 123.7 | 16.6 | .211 | 118.1 | 16.7 | .320 | 121.1 | 13.8 | .035 |

**Supplementary Table 15.** Diastolic blood pressure in mm Hg measured at eight time points as mean and standard deviation (SD) for the five study arms.

|  | Nicotine-free pouches | | | 6 mg nicotine pouches | | | 20 mg nicotine pouches | | | 30 mg nicotine pouches | | | Tobacco cigarette | | |
| --- | --- | --- | --- | --- | --- | --- | --- | --- | --- | --- | --- | --- | --- | --- | --- |
|  | Mean | SD | p-Value | Mean | SD | p-value | Mean | SD | p-value | Mean | SD | p-value | Mean | SD | p-value |
| Baseline | 75.1 | 11.4 |  | 76.6 | 11.2 |  | 76.0 | 11.6 |  | 75.2 | 7.5 |  | 76.4 | 10.2 |  |
| 5 min | 78.0 | 11.8 | .110 | 82.1 | 12.5 | .001 | 84.9 | 8.7 | .000 | 81.6 | 5.9 | .000 | 86.0 | 9.3 | .000 |
| 20 min | 79.7 | 13.2 | .067 | 83.3 | 12.1 | .001 | 81.2 | 11.6 | .003 | 83.3 | 7.3 | .000 | 83.1 | 11.3 | .000 |
| 40 min | 78.5 | 11.2 | .088 | 77.9 | 11.5 | .462 | 77.7 | 11.3 | .346 | 80.6 | 8.0 | .059 | 78.9 | 11.8 | .575 |
| 60 min | 75.4 | 12.8 | .953 | 77.7 | 16.0 | .812 | 78.9 | 13.2 | .387 | 80.9 | 9.0 | .046 | 78.2 | 13.4 | .675 |
| 90 min | 75.7 | 13.3 | .901 | 78.0 | 14.2 | .775 | 79.5 | 11.9 | .372 | 81.3 | 10.3 | .066 | 78.4 | 11.7 | .613 |
| 120 min | 78.4 | 13.7 | .496 | 76.7 | 13.1 | .988 | 74.9 | 10.3 | .715 | 80.0 | 11.9 | .140 | 76.6 | 12.8 | .952 |
| 240 min | 75.9 | 11.3 | .849 | 77.7 | 14.9 | .821 | 78.7 | 10.2 | .501 | 78.0 | 12.9 | .313 | 76.7 | 13.3 | .892 |

Parameters for arterial stiffness were measured with a MobilOGraph™ device. Results for augmentation index adjusted at HR 75 bpm (AIX@75), and total peripheral resistance/vascular resistance (TVR) are presented in Supplementary Tables 16, 17, and 18, respectively.

**Supplementary Table 16.** Augmentation index adjusted at HR 75 bpm (AIX@75) in % measured at eight time points as mean and standard deviation (SD) for the five study arms. For difference to baseline, a p-value is given.

|  | Nicotine-free pouches | | | 6 mg nicotine pouches | | | 20 mg nicotine pouches | | | 30 mg nicotine pouches | | | Tobacco cigarette | | |
| --- | --- | --- | --- | --- | --- | --- | --- | --- | --- | --- | --- | --- | --- | --- | --- |
|  | Mean | SD | p-Value | Mean | SD | p-value | Mean | SD | p-value | Mean | SD | p-value | Mean | SD | p-value |
| Baseline | 13.1 | 12.2 |  | 17.5 | 11.3 |  | 13.7 | 9.4 |  | 13.5 | 9.6 |  | 11.9 | 9.9 |  |
| 5 min | 15.3 | 11.9 | .082 | 21.3 | 10.5 | .045 | 24.3 | 9.0 | .000 | 27.5 | 6.7 | .000 | 26.0 | 9.9 | .000 |
| 20 min | 18.9 | 11.7 | .071 | 23.3 | 9.4 | .039 | 22.9 | 10.9 | .000 | 27.9 | 12.1 | .000 | 22.5 | 8.2 | .000 |
| 40 min | 17.7 | 10.1 | .115 | 16.0 | 11.8 | .626 | 18.6 | 11.5 | .059 | 25.8 | 12.5 | .003 | 19.7 | 10.8 | .096 |
| 60 min | 15.9 | 9.3 | .498 | 17.6 | 11.0 | .982 | 21.3 | 12.5 | .056 | 18.0 | 10.5 | .183 | 18.5 | 10.3 | .131 |
| 90 min | 13.4 | 12.4 | .949 | 12.5 | 12.9 | .146 | 16.3 | 13.6 | .467 | 19.1 | 10.8 | .135 | 17.9 | 9.3 | .119 |
| 120 min | 12.8 | 11.7 | .931 | 16.1 | 10.8 | .681 | 15.3 | 8.7 | .673 | 18.6 | 12.2 | .206 | 16.0 | 10.4 | .291 |
| 240 min | 17.3 | 13.9 | .344 | 15.8 | 11.4 | .656 | 19.8 | 9.9 | .033 | 19.5 | 12.1 | .055 | 16.6 | 10.7 | .209 |

**Supplementary Table 17.** Total peripheral resistance/vascular resistance (TVR) in dyn×s/cm^5^ measured at eight time points as mean and standard deviation (SD) for the five study arms. For difference to baseline, a p-value is given.

|  | Nicotine-free pouches | | | 6 mg nicotine pouches | | | 20 mg nicotine pouches | | | 30 mg nicotine pouches | | | Tobacco cigarette | | |
| --- | --- | --- | --- | --- | --- | --- | --- | --- | --- | --- | --- | --- | --- | --- | --- |
|  | Mean | SD | p-Value | Mean | SD | p-value | Mean | SD | p-value | Mean | SD | p-value | Mean | SD | p-value |
| Baseline | 1694.6 | 247.4 |  | 1599.4 | 217.6 |  | 1524.1 | 134.8 |  | 1536.1 | 170.4 |  | 1471.1 | 211.2 |  |
| 5 min | 1663.8 | 249.8 | .609 | 1732.1 | 199.2 | .005 | 1764.2 | 187.0 | .000 | 1737.5 | 138.0 | .000 | 1725.8 | 163.5 | .000 |
| 20 min | 1711.2 | 241.2 | .829 | 1761.5 | 176.5 | .013 | 1690.4 | 166.5 | .000 | 1739.2 | 114.3 | .001 | 1683.9 | 162.6 | .001 |
| 40 min | 1695.7 | 199.8 | .984 | 1552.7 | 241.5 | .508 | 1621.8 | 171.7 | .002 | 1619.7 | 176.4 | .300 | 1606.9 | 214.1 | .176 |
| 60 min | 1678.0 | 265.5 | .880 | 1639.7 | 268.2 | .475 | 1642.8 | 152.6 | .022 | 1688.6 | 213.6 | .077 | 1656.0 | 199.4 | .037 |
| 90 min | 1635.4 | 243.4 | .559 | 1652.9 | 257.6 | .531 | 1656.1 | 180.3 | .026 | 1687.2 | 182.8 | .080 | 1701.8 | 190.3 | .026 |
| 120 min | 1653.8 | 251.8 | .655 | 1630.0 | 213.0 | .661 | 1579.2 | 338.2 | .585 | 1672.9 | 214.3 | .123 | 1722.3 | 185.6 | .008 |
| 240 min | 1555.8 | 258.2 | .167 | 1563.4 | 208.5 | .619 | 1644.2 | 218.7 | .122 | 1689.1 | 189.9 | .007 | 1583.5 | 217.5 | .093 |

# 5. Side Effects

At eleven time points, the side effects head buzz, mouth or throat irritations, lightheadedness, dizziness, cold hands or feet, palpitations, headache, perspiration, nausea, urge to vomit, and salivation were assessed on a numeric rating (NRS) from 0 (no effect) to 10 (strongest effect). Scale for salivation was from 0 = lowest salivation (dry mouth) over 5 = normal salivation to 10 = highest salivation (hypersalivation). Mean scores are presented in Supplementary Table 19.

**Supplementary Table 18.** Mean scores of side effects rated on a numeric rating scale (NRS) from 0 (no effect) to 10 (strongest effect). A different scale was used for salivation (see below).

| Time point, min | 0 | 2 | 5 | 10 | 15 | 20 | 30 | 60 | 90 | 120 | 240 |
| --- | --- | --- | --- | --- | --- | --- | --- | --- | --- | --- | --- |
| Head buzz | | | | | | | | | | | |
| Nicotine-free pouch | 0.0 | 0.7 | 1.5 | 1.2 | 1.0 | 1.2 | 0.6 | 0.3 | 0.1 | 0.0 | 0.0 |
| 6 mg nicotine pouch | 0.0 | 0.9 | 1.7 | 1.5 | 1.2 | 0.8 | 0.9 | 0.5 | 0.5 | 0.5 | 0.1 |
| 20 mg nicotine pouch | 0.0 | 1.7 | 3.0 | 2.7 | 1.5 | 1.3 | 0.9 | 0.2 | 0.1 | 0.1 | 0.0 |
| 30 mg nicotine pouch | 0.0 | 2.5 | 4.5 | 3.5 | 2.7 | 2.0 | 1.4 | 0.7 | 0.5 | 0.1 | 0.0 |
| Tobacco cigarette | 0.0 | 4.8 | 2.9 | 1.5 | 0.7 | 0.4 | 0.3 | 0.1 | 0.1 | 0.2 | 0.0 |
| Mouth irritation | | | | | | | | | | | |
| Nicotine-free pouch | 0.1 | 3.7 | 4.1 | 3.3 | 2.5 | 2.2 | 0.7 | 0.1 | 0.2 | 0.1 | 0.0 |
| 6 mg nicotine pouch | 0.3 | 4.1 | 3.5 | 3.0 | 2.6 | 2.5 | 1.3 | 0.3 | 0.1 | 0.1 | 0.1 |
| 20 mg nicotine pouch | 0.4 | 5.0 | 4.7 | 3.3 | 2.3 | 2.4 | 1.7 | 0.6 | 0.3 | 0.1 | 0.2 |
| 30 mg nicotine pouch | 0.1 | 7.5 | 5.5 | 2.9 | 1.9 | 1.6 | 0.7 | 0.1 | 0.3 | 0.3 | 0.3 |
| Tobacco cigarette | 0.3 | 1.3 | 1.4 | 1.2 | 1.0 | 1.0 | 0.5 | 0.2 | 0.1 | 0.1 | 0.3 |
| Throat irritation | | | | | | | | | | | |
| Nicotine-free pouch | 0.1 | 0.4 | 0.5 | 0.4 | 0.4 | 0.6 | 0.3 | 0.3 | 0.0 | 0.0 | 0.1 |
| 6 mg nicotine pouch | 0.4 | 0.3 | 0.3 | 0.3 | 0.3 | 0.7 | 0.3 | 0.3 | 0.1 | 0.2 | 0.1 |
| 20 mg nicotine pouch | 0.3 | 0.6 | 0.7 | 0.5 | 0.7 | 0.9 | 0.9 | 0.3 | 0.3 | 0.1 | 0.1 |
| 30 mg nicotine pouch | 0.2 | 0.5 | 0.7 | 0.6 | 0.5 | 0.4 | 0.9 | 0.0 | 0.1 | 0.1 | 0.1 |
| Tobacco cigarette | 0.5 | 1.5 | 1.5 | 1.1 | 0.6 | 0.7 | 0.7 | 0.3 | 0.2 | 0.1 | 0.4 |
| Lightheadedness | | | | | | | | | | | |
| Nicotine-free pouch | 0.1 | 0.5 | 0.9 | 1.0 | 0.7 | 0.8 | 0.2 | 0.5 | 0.3 | 0.0 | 0.1 |
| 6 mg nicotine pouch | 0.2 | 0.5 | 0.6 | 0.5 | 0.5 | 0.3 | 0.1 | 0.1 | 0.1 | 0.0 | 0.0 |
| 20 mg nicotine pouch | 0.5 | 0.7 | 1.1 | 1.2 | 0.6 | 0.3 | 0.2 | 0.1 | 0.0 | 0.0 | 0.1 |
| 30 mg nicotine pouch | 0.1 | 1.2 | 1.7 | 1.9 | 1.8 | 1.1 | 1.1 | 0.6 | 0.1 | 0.0 | 0.0 |
| Tobacco cigarette | 0.6 | 2.9 | 2.4 | 1.4 | 1.3 | 1.1 | 0.6 | 0.3 | 0.3 | 0.0 | 0.5 |
| Dizziness | | | | | | | | | | | |
| Nicotine-free pouch | 0.1 | 0.1 | 0.1 | 0.3 | 0.3 | 0.2 | 0.1 | 0.0 | 0.0 | 0.3 | 0.1 |
| 6 mg nicotine pouch | 0.1 | 0.3 | 0.3 | 0.3 | 0.3 | 0.1 | 0.1 | 0.0 | 0.1 | 0.0 | 0.0 |
| 20 mg nicotine pouch | 0.1 | 0.5 | 0.5 | 0.3 | 0.3 | 0.3 | 0.2 | 0.0 | 0.0 | 0.0 | 0.0 |
| 30 mg nicotine pouch | 0.0 | 1.1 | 1.1 | 1.1 | 1.3 | 0.9 | 0.7 | 0.2 | 0.0 | 0.3 | 0.7 |
| Tobacco cigarette | 0.4 | 2.1 | 2.0 | 0.7 | 0.5 | 0.3 | 0.1 | 0.1 | 0.0 | 0.1 | 0.4 |
| Cold hands or feet | | | | | | | | | | | |
| Nicotine-free pouch | 0.6 | 0.6 | 0.5 | 0.4 | 0.3 | 0.2 | 0.3 | 0.3 | 0.2 | 0.3 | 0.3 |
| 6 mg nicotine pouch | 0.8 | 0.6 | 0.8 | 0.6 | 0.5 | 0.5 | 0.6 | 0.5 | 0.6 | 0.6 | 0.5 |
| 20 mg nicotine pouch | 0.8 | 0.8 | 0.9 | 0.9 | 0.9 | 0.8 | 0.9 | 0.6 | 0.7 | 0.6 | 0.5 |
| 30 mg nicotine pouch | 0.5 | 0.5 | 0.7 | 0.7 | 0.7 | 0.8 | 1.1 | 0.7 | 0.8 | 0.5 | 0.1 |
| Tobacco cigarette | 1.5 | 2.2 | 2.0 | 1.9 | 1.7 | 1.5 | 1.1 | 1.5 | 1.5 | 1.1 | 1.3 |
| Palpitation | | | | | | | | | | | |
| Nicotine-free pouch | 0.1 | 0.1 | 0.1 | 0.2 | 0.1 | 0.1 | 0.1 | 0.0 | 0.1 | 0.0 | 0.0 |
| 6 mg nicotine pouch | 0.3 | 0.4 | 0.4 | 0.3 | 0.2 | 0.2 | 0.1 | 0.2 | 0.1 | 0.1 | 0.1 |
| 20 mg nicotine pouch | 0.1 | 0.3 | 0.5 | 0.5 | 0.3 | 0.3 | 0.3 | 0.0 | 0.0 | 0.0 | 0.2 |
| 30 mg nicotine pouch | 0.3 | 0.5 | 0.7 | 0.5 | 0.5 | 0.5 | 0.5 | 0.1 | 0.0 | 0.0 | 0.0 |
| Tobacco cigarette | 1.1 | 0.8 | 0.7 | 0.7 | 0.4 | 0.2 | 0.2 | 0.1 | 0.1 | 0.1 | 0.1 |
| Headache | | | | | | | | | | | |
| Nicotine-free pouch | 0.3 | 0.1 | 0.2 | 0.3 | 0.3 | 0.3 | 0.2 | 0.1 | 0.2 | 0.3 | 0.1 |
| 6 mg nicotine pouch | 0.1 | 0.1 | 0.2 | 0.2 | 0.2 | 0.3 | 0.5 | 0.2 | 0.1 | 0.1 | 0.1 |
| 20 mg nicotine pouch | 0.1 | 0.2 | 0.3 | 0.5 | 0.4 | 0.1 | 0.3 | 0.1 | 0.1 | 0.1 | 0.4 |
| 30 mg nicotine pouch | 0.0 | 0.6 | 0.7 | 0.5 | 0.1 | 0.3 | 0.3 | 0.0 | 0.1 | 0.0 | 0.1 |
| Tobacco cigarette | 0.5 | 0.3 | 0.3 | 0.4 | 0.5 | 0.3 | 0.3 | 0.3 | 0.2 | 0.3 | 0.5 |
| Perspiration | | | | | | | | | | | |
| Nicotine-free pouch | 0.3 | 0.3 | 0.3 | 0.1 | 0.1 | 0.1 | 0.1 | 0.1 | 0.1 | 0.2 | 0.1 |
| 6 mg nicotine pouch | 0.1 | 0.4 | 0.3 | 0.2 | 0.2 | 0.2 | 0.2 | 0.1 | 0.1 | 0.1 | 0.1 |
| 20 mg nicotine pouch | 0.4 | 0.6 | 0.5 | 0.7 | 0.4 | 0.3 | 0.6 | 0.3 | 0.1 | 0.2 | 0.1 |
| 30 mg nicotine pouch | 0.2 | 0.6 | 1.0 | 1.0 | 0.7 | 0.7 | 0.5 | 0.3 | 0.1 | 0.2 | 0.2 |
| Tobacco cigarette | 1.1 | 0.4 | 0.6 | 0.4 | 0.5 | 0.5 | 0.6 | 0.3 | 0.3 | 0.1 | 0.1 |
| Nausea | | | | | | | | | | | |
| Nicotine-free pouch | 0.0 | 0.1 | 0.1 | 0.1 | 0.0 | 0.0 | 0.0 | 0.0 | 0.1 | 0.0 | 0.0 |
| 6 mg nicotine pouch | 0.0 | 0.1 | 0.1 | 0.1 | 0.0 | 0.1 | 0.0 | 0.0 | 0.0 | 0.0 | 0.0 |
| 20 mg nicotine pouch | 0.0 | 0.1 | 0.3 | 0.1 | 0.4 | 0.4 | 0.3 | 0.0 | 0.0 | 0.1 | 0.1 |
| 30 mg nicotine pouch | 0.0 | 0.1 | 0.5 | 1.0 | 0.9 | 0.5 | 0.9 | 0.0 | 0.1 | 0.0 | 0.0 |
| Tobacco cigarette | 0.5 | 0.1 | 0.1 | 0.1 | 0.1 | 0.0 | 0.0 | 0.1 | 0.2 | 0.0 | 0.0 |
| Urge to vomit | | | | | | | | | | | |
| Nicotine-free pouch | 0.0 | 0.0 | 0.0 | 0.0 | 0.0 | 0.0 | 0.0 | 0.0 | 0.0 | 0.0 | 0.0 |
| 6 mg nicotine pouch | 0.0 | 0.0 | 0.0 | 0.0 | 0.0 | 0.0 | 0.0 | 0.0 | 0.0 | 0.0 | 0.0 |
| 20 mg nicotine pouch | 0.0 | 0.0 | 0.0 | 0.0 | 0.0 | 0.0 | 0.0 | 0.0 | 0.0 | 0.0 | 0.0 |
| 30 mg nicotine pouch | 0.0 | 0.0 | 0.3 | 0.9 | 0.7 | 0.4 | 0.8 | 0.1 | 0.1 | 0.0 | 0.0 |
| Tobacco cigarette | 0.2 | 0.0 | 0.0 | 0.0 | 0.0 | 0.0 | 0.0 | 0.0 | 0.0 | 0.2 | 0.0 |
| Salivation, scale from 0 = lowest salivation (dry mouth) over 5 = normal salivation to 10 = highest salivation (hypersalivation) | | | | | | | | | | | |
| Nicotine-free pouch | 3.3 | 4.1 | 3.9 | 3.5 | 3.3 | 3.7 | 3.3 | 3.0 | 3.3 | 2.9 | 3.5 |
| 6 mg nicotine pouch | 4.1 | 4.5 | 4.1 | 4.0 | 3.7 | 4.5 | 3.7 | 3.9 | 3.9 | 3.9 | 3.4 |
| 20 mg nicotine pouch | 3.4 | 3.5 | 3.4 | 2.9 | 2.7 | 3.3 | 3.4 | 3.3 | 3.3 | 3.4 | 3.5 |
| 30 mg nicotine pouch | 3.6 | 4.1 | 3.3 | 3.4 | 3.3 | 3.1 | 3.7 | 3.1 | 3.2 | 2.9 | 2.9 |
| Tobacco cigarette | 4.0 | 3.6 | 3.7 | 3.1 | 2.6 | 2.6 | 2.1 | 2.9 | 2.6 | 2.6 | 2.6 |

# 6. Gas chromatography method for nicotine determination

- Injector: Splitless, 320°C
- Column: DB-ALC1 capillary column (30 m length, 320 μm inner diameter, 1.80 μm film thickness, 10 m pre-column, Agilent Technologies, Waldbronn, Germany
- Flow: constant flow, 1.2 ml/min hydrogen (purity 99.999%, Linde, Pullach, Germany)
- Oven temperature: Program started with 40°C for 2 min, followed by a 30°C/min ramp to 260°C with 10 min hold
- Detector: FID at 320°C, 35 mL/min hydrogen flow, 350 mL/min air flow, 25 mL/min nitrogen make up flow (purity 99.999%, Linde, Pullach, Germany)
